# Complex I inhibition collapses host bioenergetics and activates p53‐dependent apoptosis in *Theileria annulata* infected cells

**DOI:** 10.3389/fcimb.2026.1908136

**Published:** 2026-10-19

**Authors:** Madhusmita Subudhi, Siva Singothu, Vasundhra Bhandari, Umarani Brahma, Kuldeep Verma, Padavala Navyaka, Akash Suresh, Sakshi Singh, Paresh Sharma

**Affiliations:** 1 National Institute of Animal Biotechnology, Hyderabad, India; 2 Graduate Studies, Regional Centre for Biotechnology (RCB), Faridabad, India; 3 Department of Pharmacoinformatics, National Institute of Pharmaceutical Education and Research (NIPER), Hyderabad, India; 4 Laboratory of Infectious Diseases, Centre for DNA Fingerprinting and Diagnostics (CDFD), Hyderabad, India

**Keywords:** buparvaquone resistance, drug resistance, mitochondrial apoptosis, oxidative stress, Rotenone, *Theileria annulata*

## Abstract

**Introduction:**

Protozoan parasites continue to impose a major burden on human and veterinary health, while emerging drug resistance increasingly limits treatment options. Identifying conserved biological vulnerabilities shared across diverse parasites may facilitate the development of new therapeutic strategies. Here, we investigated whether mitochondrial electron transport represents a common metabolic vulnerability in drug-resistant protozoan pathogens.

**Methods:**

The Medicines for Malaria Venture Global Health Priority Box (GHPB) was screened against *Theileria annulata*-infected cells to identify compounds affecting parasite survival. Rotenone was evaluated using cell viability assays, flow cytometry, mitochondrial membrane potential, reactive oxygen species (ROS) detection, apoptosis assays, transcriptomic profiling, drug combination studies, and comparative bioinformatic analyses.

**Results:**

Rotenone selectively inhibited *T. annulata*-infected cells (IC_50_ = 1.30 ± 0.27 μM) while sparing uninfected cells, retained efficacy against buparvaquone-resistant cells, and showed activity against *Leishmania donovani* (IC50 = 10.12 ± 0.98 μM), *Entamoeba* histolytica (IC50 = 32.94 ± 0.27μM), and *Naegleria fowleri* (44.27 ± 4.02% mortality at 20μM). suggesting a conserved mitochondrial vulnerability across evolutionarily distinct protozoan pathogens. Mitochondrial electron transportdisruption induced membrane depolarization, elevated ROS, γH2AX-associated DNA damage, phospho-p53 (Ser15) activation, G2/M-phase arrest, and caspase-9/-3-dependent apoptosis. Transcriptomic profiling revealed oxidative and proteotoxic stress with suppression of metabolic and translational pathways. Synergism between inhibitors of mitochondrial complexes I and III further supported mitochondrial electron transport as a therapeutically exploitable pathway.

**Discussion:**

These findings reveal mitochondria‐dependent vulnerabilities as exploitable weaknesses across protozoa. Rotenone, though unsuitable for clinical use due to systemic toxicity, serves as a prototype compound that demonstrates mitochondrial metabolic vulnerability beyond lineage boundaries. By establishing mitochondrial bioenergetics as a resistance‐independent therapeutic node, this work provides a foundation for designing safer, broad‐spectrum antiparasitic agents that selectively exploit mitochondrial pathways.

## Graphical Abstract

**Figure:**
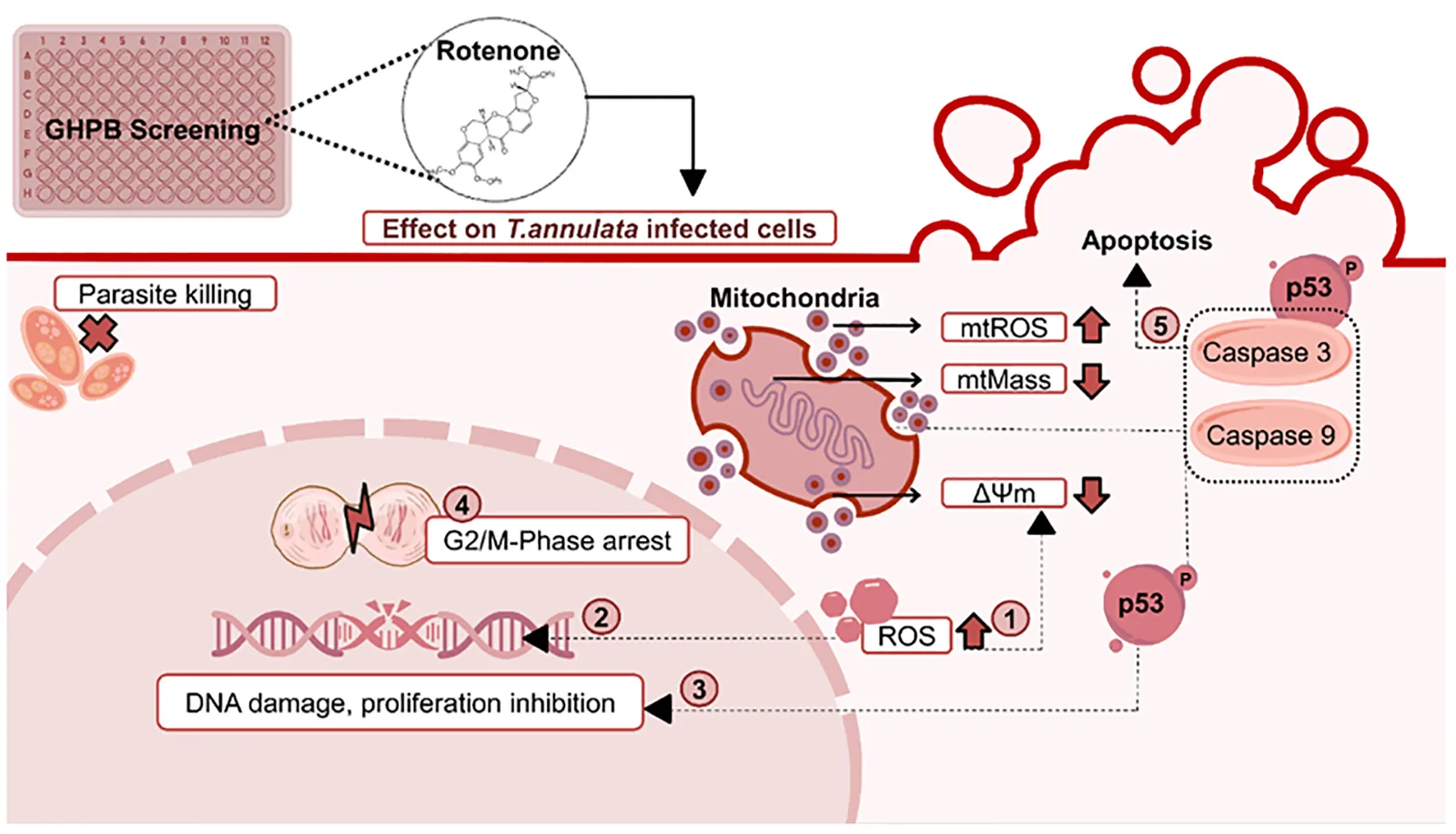
Schematic representation of Rotenone-induced cell death in Theileria annulata-infected cells. Proposed mechanism of action of Rotenone against TA cells. Screening of the Global Health Priority Box (GHPB) identified Rotenone as a potent compound that induces G2/M-phase arrest, DNA damage, and p53 activation, leading to mitochondrial dysfunction characterized by altered mtROS, mitochondrial mass, and membrane potential (ΔΨm). These events trigger the intrinsic apoptotic pathway through caspase-9 and caspase-3 activation, culminating in apoptosis of infected cells.

## Highlights

- Rotenone retains efficacy against wild-type and buparvaquone-resistant *T. annulata* infected cells, validating it as a resistance-independent therapeutic node.
- Complex I disruption triggers a temporally ordered apoptotic cascade via mitochondrial dysfunction and oxidative stress.
- Transcriptomics and functional analyses reveal a stress-collapse response underpinning Rotenone-induced cell death in infected cells.
- Cross-phylum activity of mitochondrial metabolic inhibition.

## 1 Introduction

Protozoan pathogens from diverse evolutionary lineages continue to impose significant health and economic burdens across both human and veterinary sectors (Sargsyan et al., 2025). Among these, the Apicomplexans parasite *Theileria annulata* is the causative agent of bovine theileriosis, a severe tick-borne disease endemic to tropical and subtropical regions (Agina et al., 2020). The infection leads to substantial agricultural losses due to mortality, reduced milk yield, decreased productivity, and reproductive impairments. A defining feature of *T. annulata* infection is its ability to transform bovine leukocytes into proliferative, apoptosis-resistant, cancer-like cells through parasite-driven manipulation of host NF-κB and PI3K/Akt pathways (Tajeri and Langsley, 2025). This oncogenic reprogramming not only drives systemic dissemination but also complicates therapeutic control (Valente et al., 2023).

The challenges posed by *T. annulata* represent the global health burden associated with other major protozoan pathogens, including the kinetoplastid *Leishmania donovani*, the amoebozoan *Entamoeba histolytica*, and the heterolobosean *Naegleria fowleri* (Fu et al., 2023; Kofman and Guarner, 2022; Kumar et al., 2019). Despite belonging to phylogenetically distant clades, these parasites share a common constraint: the scarcity of safe, affordable, and effective chemotherapeutic options. Drug resistance further aggravates this crisis; in bovine theileriosis, the reliance on a single approved drug, BPQ, targeting the parasite’s mitochondrial electron transport chain has led to the emergence and spread of BPQ-resistant *T. annulata* strains in endemic regions (Hacilarlioglu et al., 2025). Likewise, resistance and treatment failures are increasingly reported for other protozoan diseases, highlighting the need for therapeutics that target conserved vulnerabilities across parasite phyla (Barrett et al., 2019).

Drug repurposing offers a powerful strategy to accelerate antiparasitic drug discovery, especially when leveraging curated libraries enriched with compounds of known safety or mechanistic value. On that note, we performed a phenotypic screen of the Medicines for Malaria Venture (MMV) Global Health Priority Box (GHPB) (Adam et al., 2024), a chemically diverse collection with broad activity against neglected tropical and zoonotic pathogens. This screening approach enabled the identification of compounds with selective activity against *T. annulata*-infected cells (TA cells), and allowed us to assess potential cross-phylum activity.

From this screen, we identified Rotenone, a classical mitochondrial complex I inhibitor (Watabe and Nakaki, 2008), as a potent lead compound with efficacy not only against TA cells, including BPQ-resistant isolates, but also against *L. donovani*, *E. histolytica*, and *N. fowleri*. The discovery of a shared susceptibility to complex I inhibition across these taxonomically diverse protozoa highlights mitochondrial bioenergetics as an underexplored but promising therapeutic target. Here, we delineate the mechanistic basis of Rotenone’s activity and present evidence supporting mitochondria-focused strategies for the development of next-generation, broad-spectrum antiparasitic therapeutics.

## 2 Result

### 2.1 Phenotypic screening identifies Rotenone as a potent and selective compound against *Theileria annulata*-infected cells

To discover new chemotherapeutic agents against TA cells, we conducted a phenotypic screen of 240 structurally diverse compounds from the Medicines for Malaria Venture Global Health Priority Box (GHPB). Using resazurin-based viability assay at 10 µM, we identified 15 compounds that reduced TA cell viability by ≥40% (**Figure 1A**). Secondary validation at 2 µM confirmed three leads with potent activity: MMV002519 (Rotenone, designated VEC C04), MMV692115 (SMILES ID: {Clc1c(cc(c2nc(Nc3ccc(C(=O)NCCN(C)C)cc3)nc2)cc1)Cl} designated as ZND D04), and MMV1828058 (SMILES ID: {Fc1ccc(OC C2Nc3nc(NC4CC5(CCC C5)CC4)c(cc(C)cn6)c6n3)c2Cl} designated as ZND A06), with IC_50_ values of 1.30 ± 0.27 µM, 2.40 ± 0.03 µM, and 1.72 ± 0.43 µM, respectively (**Figure 1B**).Critically, all three compounds demonstrated selectivity for infected cells. Cytotoxicity assessment in uninfected bovine peripheral blood mononuclear cells (PBMCs) revealed >90% viability at 10 µM. Tanimoto coefficient-based structural similarity analysis confirmed low chemical overlap among the lead compounds and reference drugs buparvaquone (BPQ) and artesunate (**Figure 1C**), suggesting potential mechanisms of action distinct from existing therapies. Time-course analysis revealed that Rotenone induced the earliest effects, with observable cell death within 24 hr—preceding ZND D04, ZND A06, and BPQ (**Figures 1D, E**). Western blot analysis demonstrated dose-dependent reduction of *Theileria annulata* surface protein (TaSP) expression following treatment (**Figures 1F, G**), confirming compound-mediated suppression of parasite viability.

**Figure 1:**
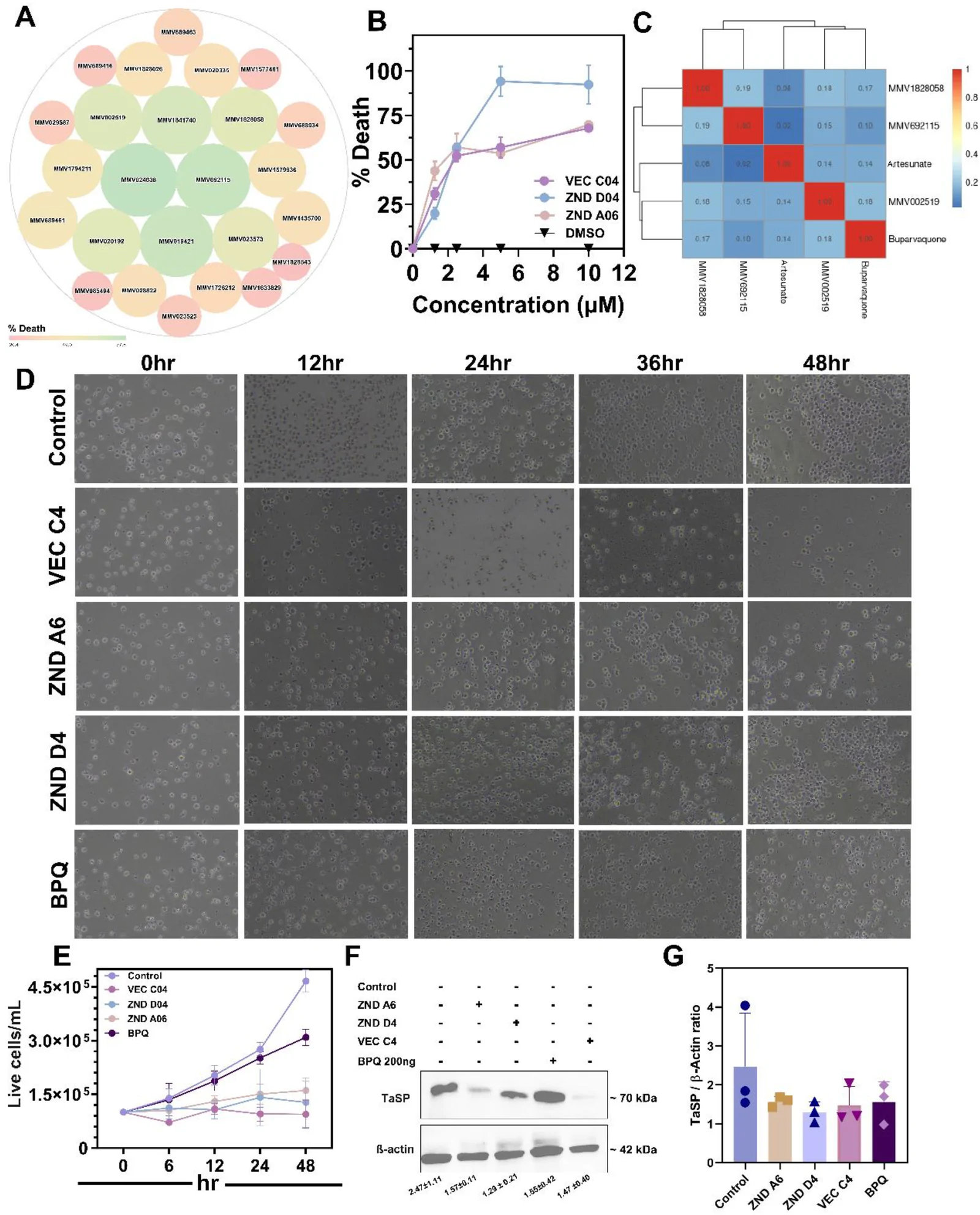
Screening and functional characterization of selected GHPB compounds. **(A)** Initial screening of the GHPB compound library at 10µM (n=2). All the Compounds inducing greater than 20% cell death are highlighted using scaled circles, where circle size corresponds to the percentage of cell death. Visualisation was performed using RAWGraphs 2.0. **(B)** Dose–response curves and calculated IC_50_ values for three lead compounds—MMV002519 (VEC C04), MMV692115 (ZND D04), and MMV1828058 (ZND A06)—tested within a 2 µM concentration range (n=2). **(C)** Structural similarity assessment based on Tanimoto coefficients for the three selected GHPB compounds in comparison to known reference compounds, Artesunate and Buparvaquone (BPQ) using ChemMine Tools. **(D)** Growth kinetics of TA cells treated with the three GHPB compounds over 72 hr, assessed at 12-hr intervals. BPQ was used as a positive control. **(E)** Quantification of growth inhibition from the kinetic assay, averaged across two biological replicates and six technical replicates per condition (n=3). **(F)** Western blot analysis of TaSP (*Theileria annulata* Surface Protein) expression in treated cells (n =3). ß-actin served as a loading control. All three compounds and BPQ (positive control) were included. **(G)** Densitometric quantification of TaSP expression normalized to ß-actin, based on three biological replicates.

### 2.2 Rotenone induces apoptosis and overcomes buparvaquone resistance

Having identified Rotenone as the most rapidly acting compound, we next characterized its apoptotic and cell cycle arrest effect on TA cells. Flow cytometric analysis of Annexin V/propidium iodide (PI)-stained cells revealed that Rotenone treatment at IC_50_ concentrations induced apoptosis in approximately 70% of infected cells after 48 hr, substantially exceeding the apoptotic responses elicited by ZND A06 (~45%) and ZND D04 (~43%) (**Figures 2A, B**).

**Figure 2:**
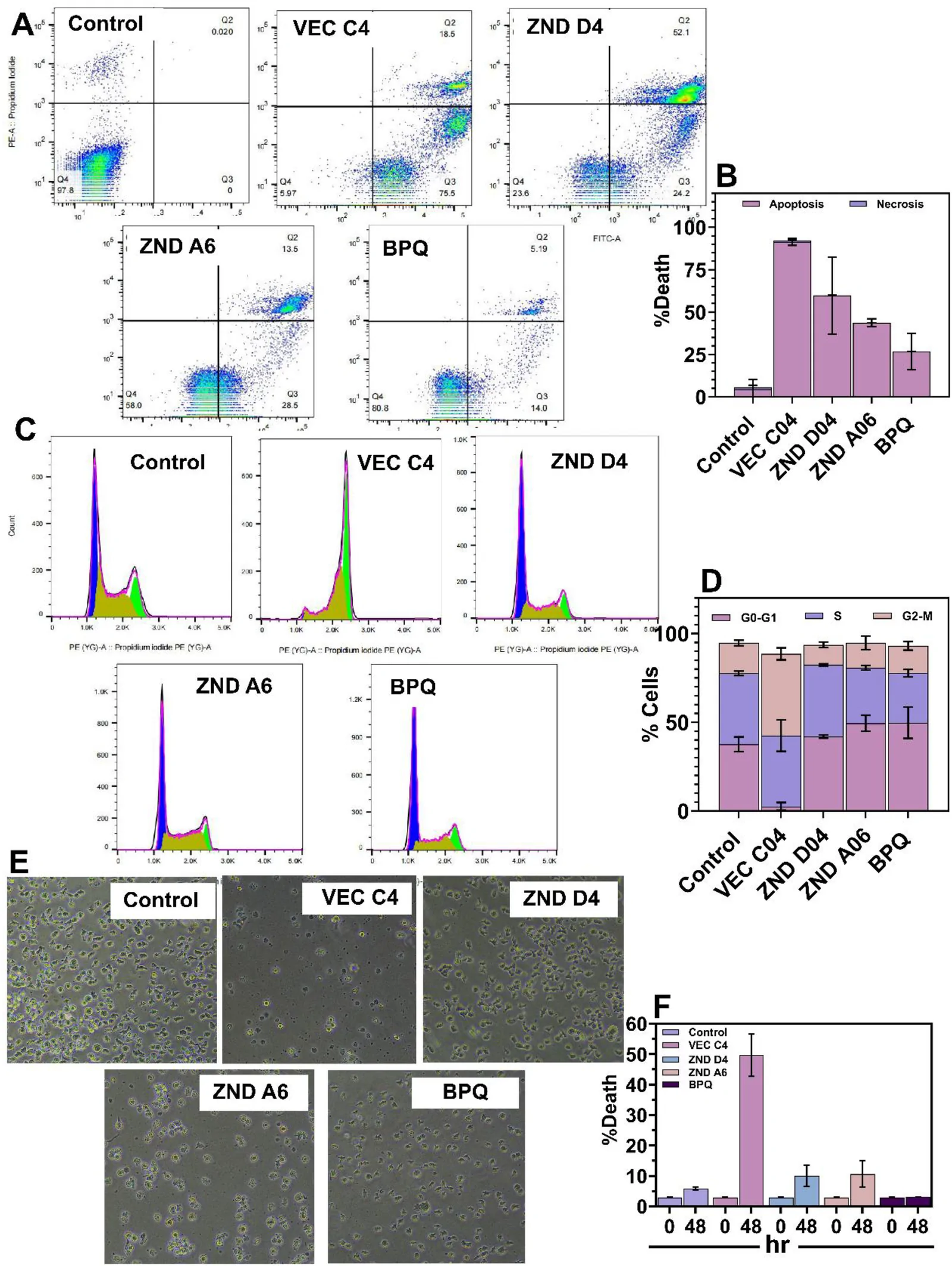
Apoptosis induction, cell cycle perturbation, and effect on BPQ-resistant cells by GHPB compounds. **(A)** Annexin V-FITC and Propidium Iodide (PI) staining of TA cells treated with GHPB compounds—VEC C04, ZND D04, ZND A06—and the reference compound Buparvaquone (BPQ, 200 ng)- all compounds treated for 48 hr, illustrating early and late apoptosis. **(B)** Quantitative analysis of apoptotic and necrotic populations based on Annexin V/PI staining in two biological replicates (n=2), with the proportion of cells in each category plotted for comparison. **(C)** Cell cycle distribution determined by PI staining. Representative histograms for control and compound-treated cells (VEC C04, ZND D04, ZND A06, and BPQ for 48hr) display alterations in G1, S, and G2/M phases. **(D)** Quantification of the cell cycle analysis showing the percentage of cells in each phase (G1, S, and G2/M), indicating compound-specific effects on cell cycle progression in two biological replicates (n=2). **(E)** Bright-field microscopic images of BPQ-resistant (BPQ-R) cells treated with VEC C04, ZND D04, ZND A06 at their IC_50_ Concentration, and BPQ 200 ng for 48 hr, demonstrating morphological changes and potential effects. **(F)** Quantitative assessment of cell death in BPQ-R cells following treatment, measured using the trypan blue exclusion assay. The data represent the percentage of non-viable cells across treatments in two biological replicates (n=2).

Cell cycle analysis further distinguished Rotenone from the other leads. While ZND A06 and ZND D04 induced G1-phase arrest, Rotenone triggered accumulation of cells in G2/M phase (**Figures 2C, D**), indicating a distinct mechanism involving mitotic disruption.

To evaluate therapeutic relevance in the context of emerging drug resistance, we generated a BPQ-R cells through stepwise drug exposure (Singh et al., 2025). When tested against this resistant line, ZND A06 and ZND D04 showed minimal activity, suggesting shared resistance mechanisms with BPQ. In contrast, Rotenone retained potent efficacy, inducing approximately 50% cell death in BPQ-R cells (**Figures 2E, F**). Since Rotenone worked against BPQ-R cells, we did its cytotoxicity assay from 100µM. Rotenone showed no detectable toxicity even at 100 µM in PBMCs establishing a favourable therapeutic window. These findings suggest that Rotenone acts through a pathway mechanistically distinct from BPQ-mediated complex III inhibition and positions it as a valuable tool for overcoming existing resistance.

### 2.3 Rotenone activates intrinsic apoptotic signaling through DNA damage and p53 engagement

To delineate the molecular pathway underlying Rotenone-induced apoptosis, we examined hallmark events of programmed cell death. TUNEL assays revealed a significant increase in DNA fragmentation within 24 hr of treatment, a defining feature of late-stage apoptosis (**Figures 3A, B**). DNA damage typically activates p53-dependent apoptotic signalling. Immunofluorescence analysis confirmed marked phosphorylation of p53 at serine 15 in Rotenone-treated cells, indicating engagement of the DNA damage response (**Figures 3C, D**). To identify the specific apoptotic pathway activated, we assessed caspase cleavage by Western blotting. Rotenone treatment elevated levels of cleaved caspase-9 and caspase-3, while cleaved caspase-8 remained unchanged (**Figure 3G**). This pattern, confirmed by immunofluorescence showing enhanced caspase-9 localisation (**Figures 3E, F**), demonstrates selective activation of the intrinsic (mitochondrial) apoptotic pathway. DAPI staining revealed characteristic nuclear condensation in treated cells, providing additional morphological evidence of apoptosis (**Figure 3H**). Collectively, these findings establish that Rotenone induces apoptosis in TA cells through a coordinated sequence: G2/M-phase arrest, DNA damage/cellular stress, p53 activation, caspase-9, caspase-3, and apoptosis.

**Figure 3:**
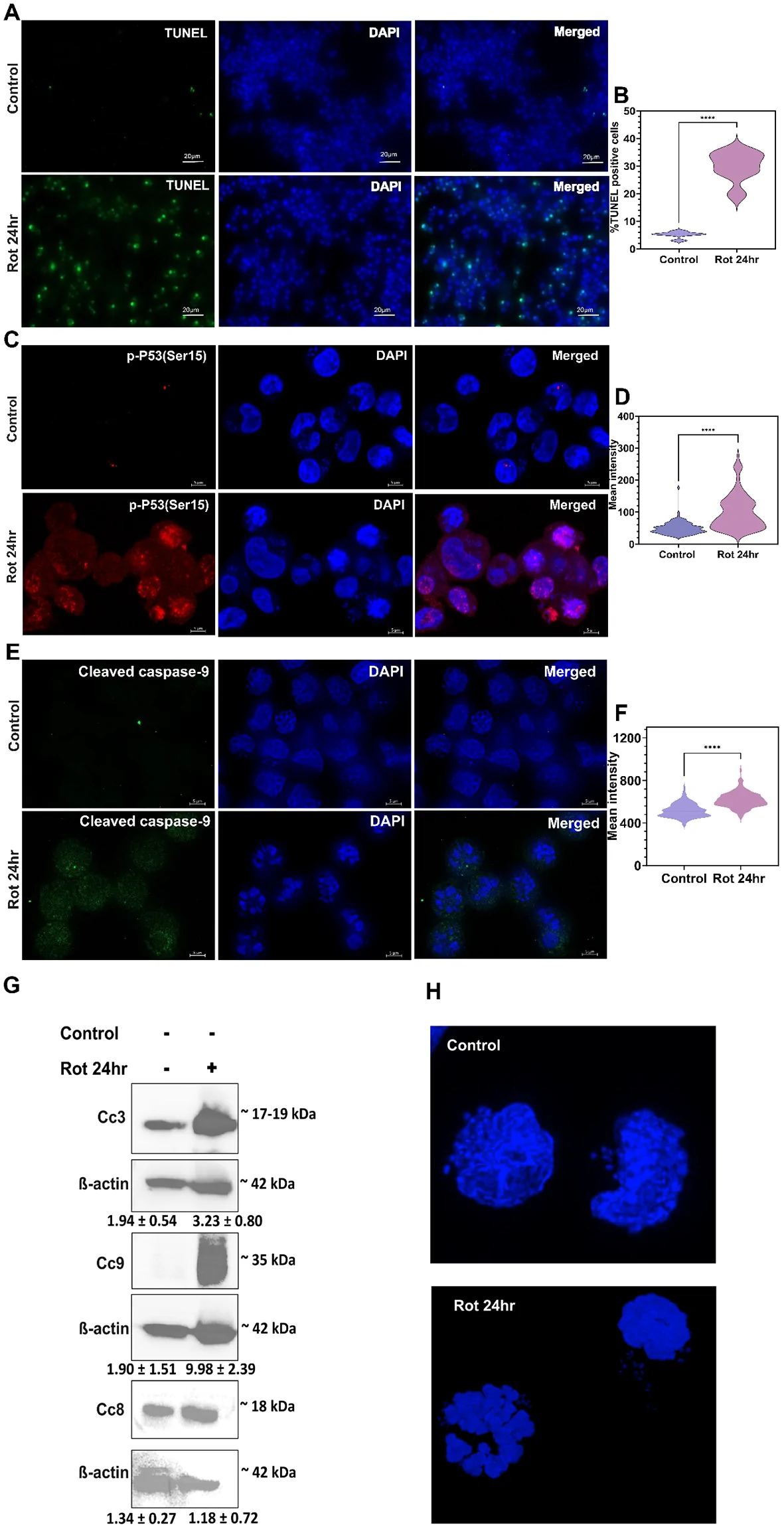
Rotenone induces DNA fragmentation and activates apoptosis-associated signaling pathways in TA cells. **(A)**TUNEL assay detecting DNA fragmentation in control and Rotenone-treated cells after 24 hr. Apoptotic nuclei exhibiting DNA breaks are visualized via fluorescence microscopy. **(B)** Quantification of TUNEL-positive cells reveals a significant increase in apoptotic cells following Rotenone treatment (unpaired *t*-test, ****p < 0.0001). **(C)** Immunofluorescence analysis of phosphorylated p53 (Ser15) in control and Rotenone-treated cells. p53 is shown in red; nuclei are counterstained with DAPI (blue). **(D)** Quantitative analysis of p53 (Ser15) fluorescence intensity shows significant elevation upon Rotenone treatment (unpaired *t*-test, ****p < 0.0001). **(E)** Detection of cleaved Caspase-9 in control and Rotenone-treated cells using immunofluorescence. Caspase-9 is labeled in green; DAPI marks the nuclei. **(F)** Fluorescence quantification demonstrates a robust increase in cleaved Caspase-9 signal after Rotenone treatment (unpaired *t*-test, ****p < 0.0001). **(G)** Western blot analysis of cleaved Caspases-3, -8, and -9 in control and Rotenone-treated samples at 24 hr (n=3). β-actin serves as a loading control. Densitometric analysis was performed using ImageJ. **(H)** Confocal microscopy (Oxford spinning disk) images of DAPI-stained nuclei illustrate nuclear condensation and fragmentation in Rotenone-treated cells compared to control, consistent with apoptotic morphology.

### 2.4 Transcriptomic profiling reveals Rotenone-induced metabolic collapse and stress adaptation

*T. annulata* transforms host leukocytes into proliferative, cancer-like cells by hijacking metabolic pathways. To determine how Rotenone disrupts this transformation, we performed RNA sequencing on infected leukocytes following 48 hr of treatment. Differential expression analysis identified 26 upregulated and 36 downregulated genes (FDR< 0.05, |log_2_FC| ≥ 1.0), revealing a coordinated stress response coupled with suppression of biosynthetic and proliferative programs (**Figure 4A**).

**Figure 4:**
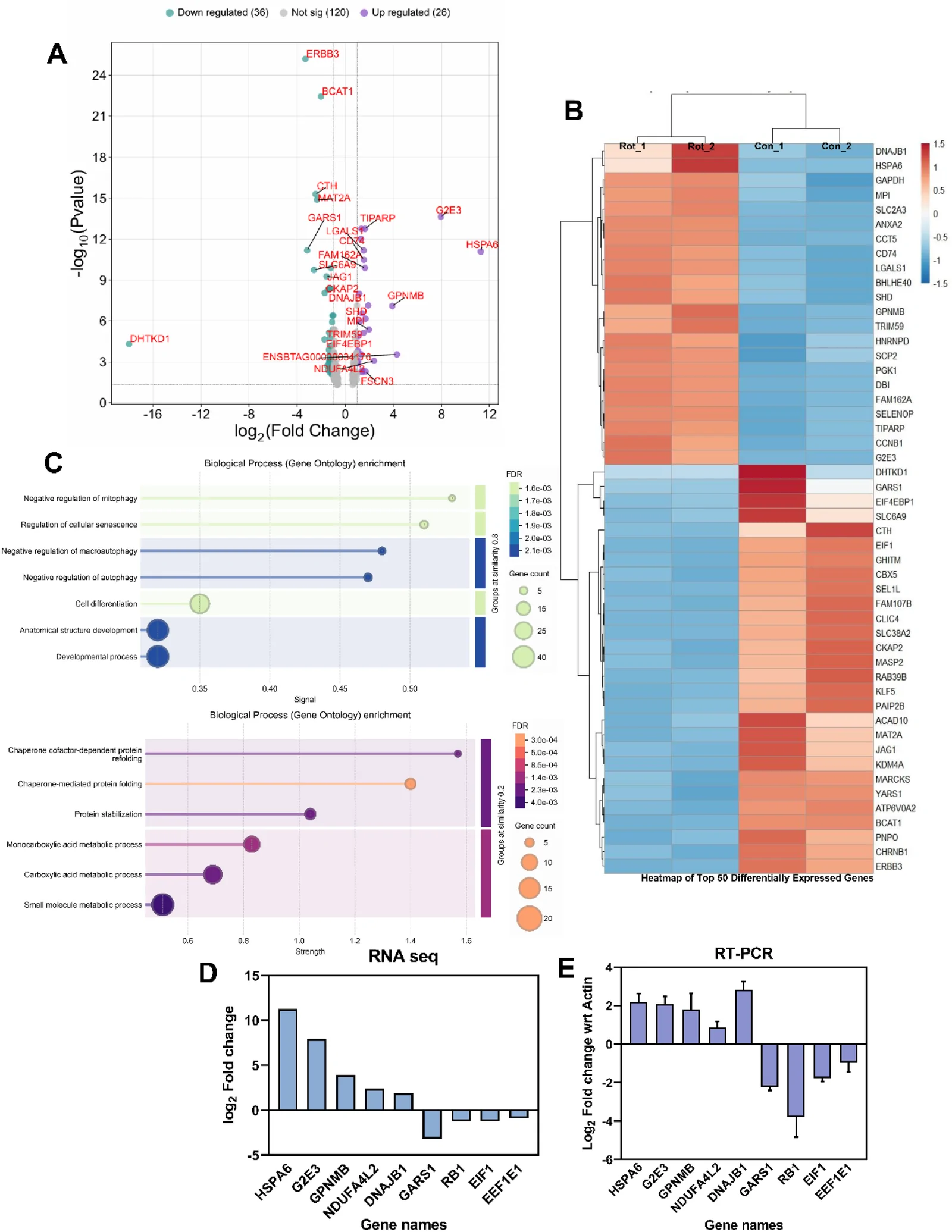
Transcriptomics analysis identifies key gene expression alterations and enriched biological processes following Rotenone treatment. **(A)** Volcano plot illustrating differentially expressed genes (DEGs) in Rotenone-treated versus control cells at 48 hr (n = 2 biological replicates per group). The X-axis shows log_2_ fold change, and the Y-axis represents –log_10_ p-values. Genes exhibiting significant upregulation and downregulation are highlighted. **(B)** Heatmap displaying the top 50 most significantly upregulated and downregulated genes in response to Rotenone exposure. A color scale from orange (+1.5 log_2_ fold) to blue (–1.5 log_2_ fold) represents normalized expression levels across individual samples. **(C)** Gene Ontology (GO) enrichment analysis of upregulated transcripts performed using STRING database reveals enrichment in biological processes related to cellular stress responses, DNA damage repair, and apoptotic signaling pathways. **(D)** RNA-seq analysis showing the log_2_ fold change in expression of selected differentially expressed genes identified from the transcriptomic analysis. Genes displaying positive and negative log_2_ fold changes represent upregulation and downregulation, respectively. **(E)** RT-qPCR validation of the selected differentially expressed genes identified by RNA-seq. The relative transcript levels are presented as log_2_ fold change, with error bars representing the variation among 3 biological replicates (n=3). The RT-qPCR results showed a consistent direction of expression change with the RNA-seq data for the selected genes.

Among the upregulated transcripts, HSPA6 (Chand et al., 2021)showed the largest positive fold change in the RNA-seq dataset (log2FC = +11.28), Additional stress-responsive transcripts included G2E3(+7.95), a p53-regulated E3 ubiquitin ligase involved in DNA damage responses (Brooks et al., 2008), GPNMB (+3.92), a lysosomal glycoprotein linked to ROS generation (Nishida et al., 2025), NDUFA4L2 (+2.39), which attenuates oxidative phosphorylation under stress (Liu et al., 2021), along with the co-chaperone DNAJB1 (+1.91), indicated adaptive responses to mitochondrial dysfunction (Kim and Hong, 2022), and other stress-responsive genes. (**Figure 4B**). These changes were consistent with activation of proteotoxic, DNA-damage-associated, oxidative-stress, and mitochondrial adaptive responses. Conversely, GARS1(−3.17), required for translational fidelity (Boczonadi et al., 2018), RB1(−1.21) a tumour suppressor (Dyson, 2016), EIF1, and EEF1E1 (**Figure 4B**) were among the downregulated genes, suggesting suppression of translational and proliferative programs (Grosely et al., 2025). Gene ontology enrichment represented processes related to glycolysis, oxidative stress responses, and cell cycle modulation (**Figure 4C**).

Because the RNA-seq analysis was performed with only two biological replicates per condition, these results were interpreted cautiously and treated primarily as a discovery-oriented dataset. To independently evaluate the reproducibility of the principal transcriptional signatures, selected genes were analysed by RT-qPCR using three independent biological replicates. The direction of differential expression observed by RNA-seq was confirmed for HSPA6, G2E3, GPNMB, NDUFA4L2, DNAJB1, GARS1, RB1, EIF1, and EEF1E1, supporting the major pathway-level patterns identified by RNA-seq (**Figures 4D, E**). By contrast, DHTKD1 showed marked down regulation in the RNA-seq dataset (log2FC = -17.98), but this change was not consistently reproduced by independent RT-qPCR analysis and was therefore not considered a validated gene-level response.

### 2.5 Functional validation confirms mitochondrial dysfunction, oxidative stress, and proliferative arrest

To further investigate the biological processes highlighted by transcriptomic profiling, we assessed mitochondrial function, oxidative stress, DNA damage, and proliferative capacity using complementary biochemical and cellular approaches.

JC-1 staining revealed that untreated infected cells maintained a high red-to-green fluorescence ratio (~2.5), indicative of intact mitochondrial membrane potential. Rotenone treatment for 48 hr reduced this ratio to ~0.88, demonstrating significant depolarization (**Figures 5A, B**). MitoTracker Green staining corroborated reduced mitochondrial mass and activity (**Figures 5C, D**). MitoSOX staining revealed progressive mitochondrial ROS accumulation, showing marked increase by 48 hr of drug treatment (**Figures 5E, F**). We examined the contribution of oxidative stress to the Rotenone-induced phenotype using the ROS scavenger N-acetylcysteine (NAC). Treatment with Rotenone for 24 hr increased DCFDA fluorescence from a mean intensity of 35, 259 in control cells to 68, 357.5, corresponding to an ~1.94-fold increase. Treatment with 40 µM NAC in combination with Rotenone reduced the DCFDA fluorescence to 42, 981, representing an approximately 37.1% reduction compared with Rotenone treatment alone. NAC treatment alone produced a DCFDA fluorescence intensity of 33, 336.5, comparable to that observed in control cells. These findings indicate that NAC attenuates Rotenone-induced ROS accumulation and support a functional contribution of oxidative stress to the cellular effects of Rotenone, (**Figures 5G, H**), validating the transcriptome-predicted oxidative stress response. Elevated ROS correlated with genomic damage. Both immunofluorescence microscopy and Western blotting demonstrated increased γH2AX expression a marker of DNA double-strand breaks, after 48 hr of treatment (**Figures 6A–C**). Consistent with *HSPA6* upregulation, Western blot analysis showed increased HSP70 protein levels beginning at 24 hr (**Figures 6D**). To further investigate the effects of Rotenone on mitochondrial function, intracellular ATP levels were measured following 24 hr treatment with Rotenone. Control cells exhibited an ATP content of 32.33 ± 3.19 nmol/10^6^ cells, whereas Rotenone-treated cells showed a marked reduction in ATP content to 7.21 ± 0.69 nmol/10^6^ cells. These findings indicate substantial impairment of cellular energy metabolism following Rotenone exposure (**Figure 6E**).

**Figure 5:**
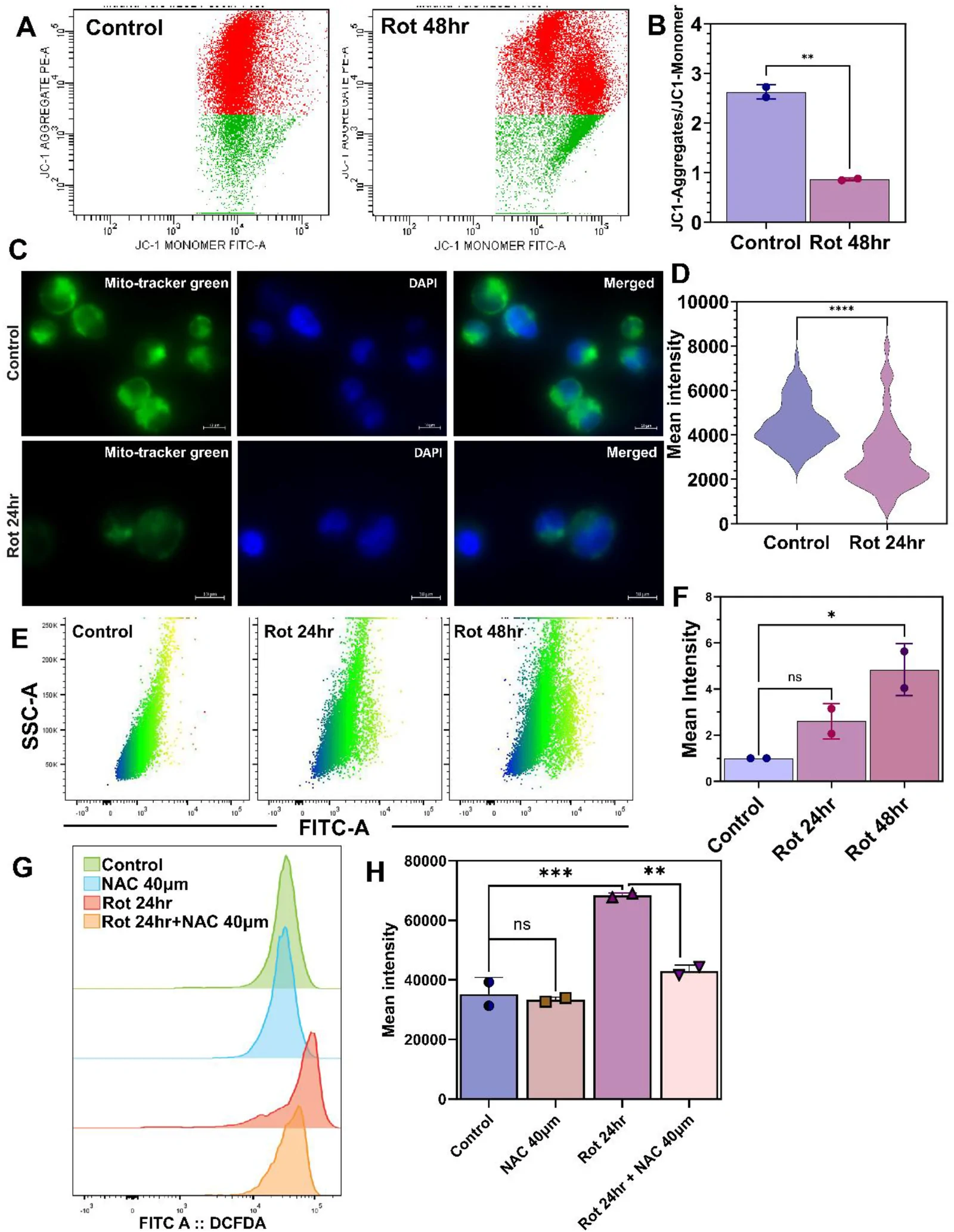
Rotenone disrupts mitochondrial membrane potential and mass and induces mitochondrial and intracellular ROS generation in TA cells. **(A)** JC-1 assay depicting mitochondrial membrane potential in control and Rotenone-treated (48 hr) cells. Green fluorescence indicates JC-1 monomers (depolarised mitochondria), which increase upon Rotenone treatment, whereas red fluorescence indicates JC-1 aggregates (polarised mitochondria), which are prominent in control cells. **(B)** Quantification of JC-1 red-to-green fluorescence ratio, showing significant mitochondrial depolarization in Rotenone-treated cells in two biological replicates (n=2). (p< 0.0034, unpaired t-test). **(C)** MitoTracker Green staining at 24 hr post-treatment, showing mitochondrial mass in control and Rotenone-treated cells. Green: mitochondria; blue: DAPI nuclear staining. **(D)** Quantitative analysis of MitoTracker intensity reveals significant reduction in mitochondrial mass signal in Rotenone-treated cells in two biological replicates (n=2) **** p< 0.0001, unpaired t-test). **(E)** MitoSOX Red assay analyzing mitochondrial ROS levels at 0 hr, 24 hr, and 48 hr post-Rotenone treatment. Increased green-shift in fluorescence indicates elevated ROS generation. X-axis: FITC-A; Y-axis: SSC-A. **(F)** Quantification of MitoSOX fluorescence intensity using two-way ANOVA shows significant time-dependent ROS induction in two biological replicates (n=2). **(G)** Representative DCFDA fluorescence histograms showing intracellular ROS levels in control, NAC (40 µM)-treated, Rotenone (24 hr)-treated, and Rotenone + NAC (40 µM)-treated cells. **(H)** Quantification of DCFDA mean fluorescence intensity showing increased ROS generation following Rotenone treatment and attenuation of this increase by NAC co-treatment. (n=2).

**Figure 6:**
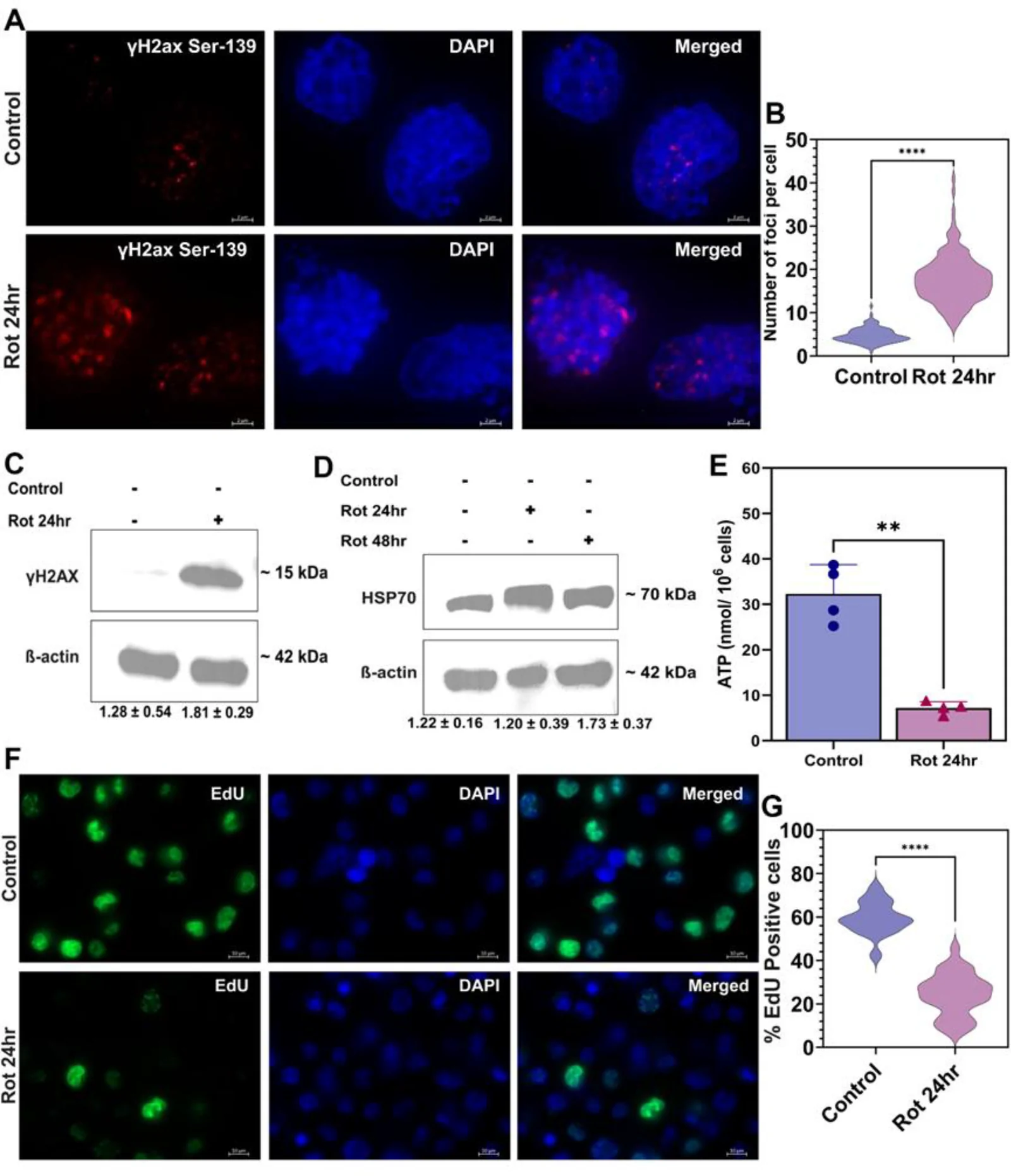
Rotenone-induced mitochondrial dysfunction is associated with DNA damage, cellular stress, and impaired proliferation in TA cells. **(A)** Immunofluorescence assay (IFA) showing phosphorylated γ-H2AX (Ser139) foci per cell (red) indicating DNA damage in Rotenone-treated cells versus control; blue: DAPI. **(B)** Quantification of γ-H2AX foci per cell demonstrates significantly increased DNA damage following Rotenone exposure (n=2). **(C)** Western blot analysis of γ-H2AX (Ser139) levels in control and Rotenone-treated (24 hr) cells. Densitometric quantification shows increased γ-H2AX expression post-treatment (n=3), normalized to β-actin. **(D)** Western blot of HSP70 expression in control, Rotenone 24 hr, and 48 hr treated cells, showing elevated HSP70 levels over time, normalized to β-actin (n=3). **(E)** ATP content measured in control and Rotenone-treated cells after 24 h exposure. Rotenone significantly reduced cellular ATP levels (32.33 ± 3.19 vs. 7.21 ± 0.69 nmol/10^6^ cells (n = 4; paired t-test, p< 0.01). Data are mean ± SD. **(F)** EdU incorporation assay assessing cell proliferation. Green: EdU-positive cells; blue: DAPI. Rotenone-treated cells show reduced proliferation at 24 hr. **(G)** Quantification of % EdU-positive cells demonstrates significant reduction in DNA synthesis upon Rotenone treatment (n=2). All data are presented as mean ± SD, with individual points representing biological replicates. Statistical significance was determined using the appropriate statistical test of an unpaired t-test. ns, not significant; *p< 0.05; **p< 0.01; ***p< 0.001; ****p< 0.0001.

EdU incorporation assays revealed marked reduction in DNA synthesis as early as 24 hr post-treatment (**Figures 6F, G**), aligning with transcriptomic downregulation of *RB1*, *ERBB3*, *GARS1*, *EIF1*, and *EEF1E1*. These functional data confirm that Rotenone disrupts mitochondrial function, generates oxidative stress, induces DNA damage, and arrests proliferation, converging on mitochondrial pathway-mediated apoptosis in the TA cells.

### 2.6 Combination therapy and additional inhibitors validate mitochondrial complex-I as a therapeutic target

Having established Rotenone’s mechanism, we investigated whether combining mitochondrial inhibitors could enhance efficacy and further validate the electron transport chain as a targetable vulnerability. Fractional inhibitory concentration (FIC) index analysis with the help of checkerboard assay revealed synergistic interactions across tested combinations. Rotenone combined with BPQ (complex III inhibitor) yielded the strongest synergy (FIC = 0.2389 ± 0.12), followed by Rotenone with atovaquone (ATQ) (FIC = 0.433 ± 0.084) and ATQ with BPQ (FIC = 0.436 ± 0.0206). The ATQ + BPQ combination, targeting the same complex, showed comparatively moderate synergy, consistent with overlapping mechanisms (**Figure 7A**). To further characterize these interactions using a multidimensional dose–response framework, the same combination datasets were analysed using SynergyFinder, applying the Highest Single Agent (HSA) model (Ianevski et al., 2022). Using the Highest Single Agent (HSA) model, synergy scores were 11.58 for Rotenone + BPQ, 5.48 for ATQ + BPQ, and 1.87 for Rotenone + ATQ. The Loewe additivity model yielded a score of 5.51 for ATQ + BPQ, reflecting their shared target. Positive scores across models indicate that combinations targeting distinct ETC nodes (complex I + complex III) produce enhanced effects compared to single agents (**Figure 7B**). Thus, although the checkerboard analysis indicated synergistic interactions for the tested combinations, the SynergyFinder analysis revealed that the magnitude and direction of the interaction depended on the reference model used. These findings highlight the importance of evaluating drug interactions using complementary analytical approaches rather than relying on a single synergy model (**Supplementary Figures 1A–D**).

**Figure 7:**
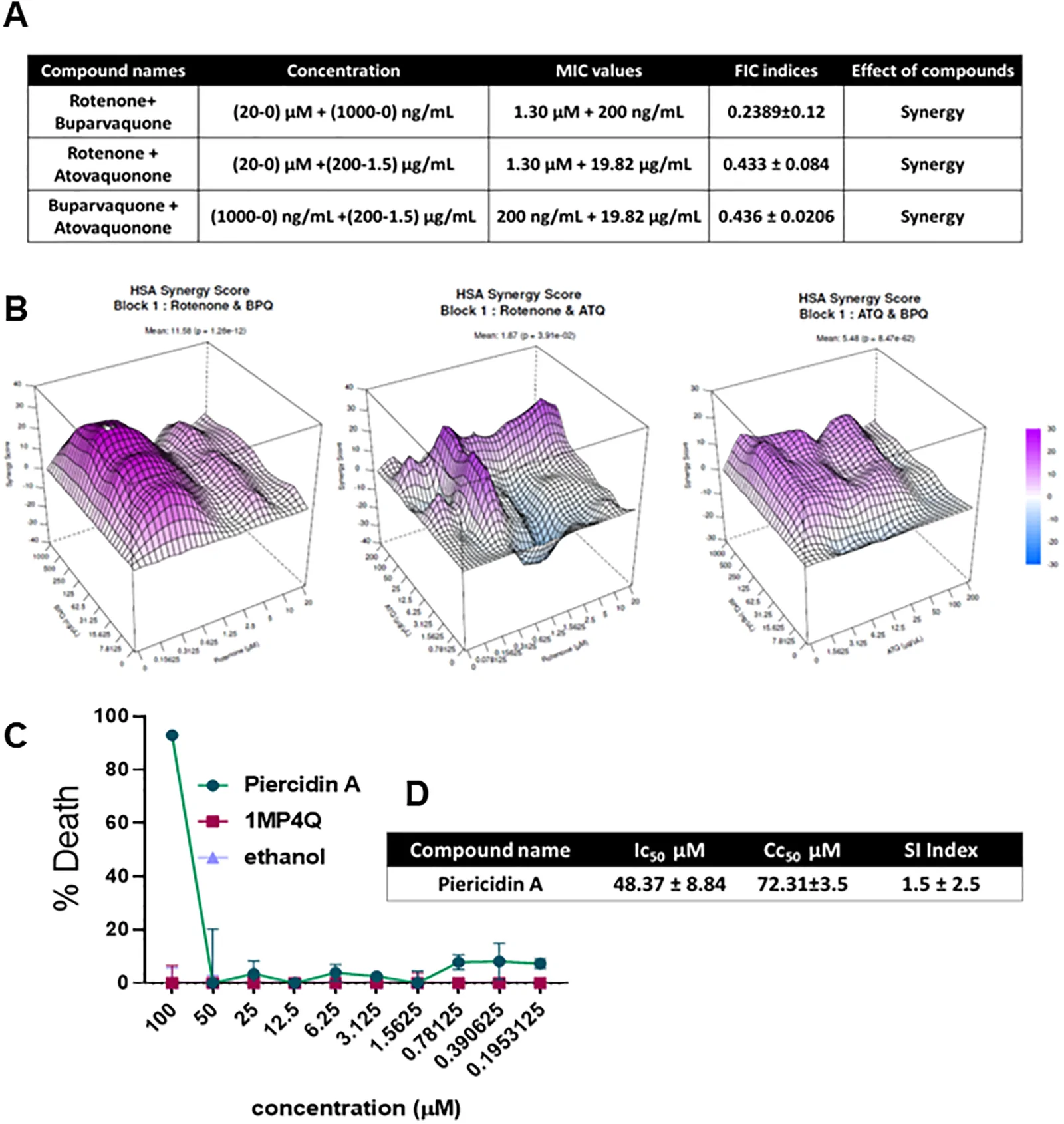
Integration of combination therapy and mitochondrial target validation in TA cells. **(A)** Drug interaction analysis based on fractional inhibitory concentration (FIC) indices for combinations of Atovaquone (ATQ), Buparvaquone (BPQ), and Rotenone (ROT). All tested combinations: ATQ + ROT, BPQ + ROT, and ATQ + BPQ demonstrated synergistic interactions (FIC< 0.5), with the strongest synergy observed for BPQ + ROT. Combinations targeting distinct mitochondrial complexes (I and III) showed greater synergy compared to those targeting the same complex (n=3). **(B)** Synergy landscape analysis using SynergyFinder across full dose–response matrices. Heatmaps and 3D response surfaces illustrate synergy scores calculated using the Highest Single Agent (HSA). Positive synergy scores across models indicate cooperative drug interactions. ATQ + BPQ showed moderate synergy consistent with shared targeting of mitochondrial complex III, whereas combinations involving ROT demonstrated variable but enhanced synergy depending on concentration. **(C)** Evaluation of additional mitochondrial inhibitors in TA cells. Dose–response curves showing the effects of Piericidin A and 1-Methyl-2-pentyl-4(1H)-quinolinone (1MPQ). Piericidin A exhibited moderate inhibitory activity, while 1MPQ showed no significant effect under the tested conditions. Ethanol served as the vehicle control. **(D)** Quantitative analysis of Piericidin A activity, indicating IC_50_ and CC_50_ values, reflecting moderate potency with limited selectivity toward infected cells (n=2).Data are mean ± SD.

To assess whether other complex I inhibitors recapitulate Rotenone’s effects, we tested Piericidin A and 1-methyl-2-pentyl-4(1H)-quinolinone. Piericidin A demonstrated measurable activity (IC_50_ = 48.37 ± 8.84 µM; CC_50_ = 75.85 ± 2.84 µM), albeit with limited selectivity. Accordingly, this data should be interpreted with caution and do not provide evidence that Piericidin A acts as a selective pharmacological probe for NADH dehydrogenase/Complex I in TA cells. In contrast, 1-methyl-2-pentyl-4(1H)-quinolinone showed no detectable inhibition (**Figures 7C, D**). These findings indicate that while complex I inhibition might have the capability to show its effect TA cells, sensitivity varies among inhibitors, highlighting the need for optimized scaffolds with improved potency and selectivity.

### 2.7 Rotenone shows preliminary cross-phylum activity against phylogenetically diverse protozoan pathogens and reveals a putative mitochondria-associated structural correlate

Rotenone is classically understood as a selective inhibitor of mammalian-type mitochondrial Complex I (NADH: ubiquinone oxidoreductase). Protozoan parasites, however, are widely described as lacking this Rotenone-sensitive complex, instead relying on the alternative type II NADH dehydrogenase (NDH2) (Lin et al., 2009), an enzyme considered Rotenone-insensitive. This raises an important question: how does Rotenone exert antiprotozoal activity against organisms that ostensibly lack its canonical target? Given that prior studies had reported activity of Rotenone and its derivatives against *Plasmodium* and *Leishmania* species (Upegui et al., 2014), we sought to systematically evaluate its activity across phylogenetically diverse protozoan pathogens and to investigate the mechanistic basis of this broader spectrum.

To this end, we tested Rotenone against three pathogens occupying distinct evolutionary branches: the kinetoplastid *Leishmania donovani*, the amoebozoan *Entamoeba histolytica* and the heterolobosean *Naegleria fowleri* (**Supplementary Figure 2A**). Rotenone demonstrated potent activity against *L. donovani* promastigotes (IC_50_ = 10.12 ± 0.98 µM) and moderate activity against *E. histolytica* (IC_50_ = 32.86 ± 0.32 µM). In *N. fowleri*, Rotenone treatment at 20 µM resulted in 44.27 ± 4.02% mortality across three independent experiments. The SI index is provided in **Supplementary Figure 2B**. The behaviour of *E. histolytica* provides the critical mechanistic control. This organism has lost canonical mitochondria entirely, retaining only mitosomes, vestigial organelles that lack a respiratory chain (Santos and Nozaki, 2022).

This mechanistic interpretation prompted a deeper structural question: if Rotenone’s activity tracks with the presence of functional mitochondria, and if the parasites in question use NDH2 rather than canonical Complex I, could the conserved architecture of NDH2 itself constitute a shared structural vulnerability? To address this, we performed comparative sequence alignment, conserved motif discovery, and phylogenetic analysis of NDH2 orthologs across the protozoan parasites (**Supplementary Figures 3A–C**).

The analysis revealed a striking degree of structural coherence beneath the surface of evolutionary divergence. Shared sequence motifs and a common PTZ00318 superfamily domain architecture were identified across *Plasmodium*, *Theileria*, *Babesia*, *Toxoplasma*, *Leishmania*, and *Naegleria*. *Entamoeba* was, once again, the outlier: its NDH2 ortholog showed clear divergence from this conserved pattern, consistent with its broader loss of conventional mitochondrial machinery and correlating with its reduced susceptibility to Rotenone (**Supplementary Figure 3D**). Taken together, these findings reframe what initially appears as a mechanistic paradox. Rotenone’s broad-spectrum activity does not require direct inhibition of a canonical Complex I homolog in each organism. Instead, it exploits a convergence of mitochondria-dependent vulnerabilities — ΔΨm disruption, ROS induction, and metabolic dependency on mitochondrial function that are shared across phylogenetically diverse but mitochondrion-bearing protozoa. The conservation of NDH2 architecture across susceptible organisms, and its divergence from *Entamoeba*, provides a structural correlation for the observed activity spectrum and identifies the PTZ00318 domain as a candidate target warranting further structural and functional investigation. Sequence and motif analyses identified NDH2 as a potential candidate target that may contribute to the susceptibility of selected protozoan parasites to mitochondrial Complex I inhibition. However, this hypothesis requires functional and structural validation.

## 3 Discussion

Phenotypic screening of the MMV GHPB compounds identified Rotenone, MMV692115, and MMV1828058 as potent, selective, and rapidly acting inhibitors of TA cell viability (Retore et al., 2024).This unbiased approach is particularly powerful for discovering cross-pathogen activity, as it requires no prior knowledge of molecular targets. The emergence of a classical complex I inhibitor from this screen is mechanistically significant and points to a broader principle: mitochondrial complex I represents a conserved, exploitable vulnerability across phylogenetically distant protozoa. It is important to emphasize that we do not propose Rotenone as a therapeutic candidate. Its systemic toxicity profile precludes direct clinical translation (Xu et al., 2025). Instead, Rotenone serves here as a chemical probe—a mechanistic tool that enables precise interrogation of mitochondrial vulnerability in TA cells and, more broadly, across protozoan parasites. By framing it as a probe rather than a drug, this study defines how electron transport chain (ETC) perturbation induces selective cell death, generating insights that can guide the design of safer, more selective next-generation compounds.

Within TA cells, our data delineate a temporally ordered, three-tiered mechanism. Complex I inhibition collapses mitochondrial membrane potential (ΔΨm), impairing oxidative phosphorylation, depleting ATP, and generating acute metabolic stress. This bioenergetic insult drives electron leak and superoxide generation, producing a pronounced surge in mitochondrial and cytosolic reactive oxygen species (ROS) consistent with established consequences of complex I blockade (Yang et al., 2023). The resulting oxidative burden inflicts genomic damage, evidenced by γH2AX induction, and activates p53-dependent DNA damage signaling, culminating in G2/M-phase arrest and engagement of the intrinsic apoptotic cascade through caspase-9 and caspase-3 activation. The absence of caspase-8 involvement confirms specificity for the mitochondrial pathway rather than the death receptor pathway. This cascade mirrors Rotenone’s reported effects in cancer cells (Sun et al., 2014), underscoring the shared metabolic dependencies between TA cells and cancer cells, and highlighting the potential of mitochondrial-targeting strategies to reverse parasite-induced transformation.

Transcriptomics data provide a view of how infected cells respond to complex I inhibition. The transcriptional landscape is dominated by a dual response: an acute proteotoxic stress program coupled with irreversible suppression of biosynthetic and proliferative pathways. Upregulation of *HSPA6*, *DNAJB1*, *G2E3*, *GPNMB*, and *NDUFA4L2* reflects cellular attempts to manage misfolded proteins, contain ROS-mediated damage, and adaptively suppress mitochondrial respiration (Brooks et al., 2008; Chand et al., 2021; Kim and Hong, 2022; Liu et al., 2021; Nishida et al., 2025; Shorbagi and Brown, 2016). This stress adaptation is insufficient, however, to offset the simultaneous downregulation of *ERBB3*, *RB1*, *GARS1*, and multiple translational regulators—genes that collectively drive bioenergetic failure, halt proliferation, and dismantle protein synthesis machinery (Black et al., 2019; Dyson, 2016; Liu et al., 2024). A particularly consequential finding is the retained potency of Rotenone against BPQ-resistant TA cells. BPQ targets parasite-encoded cytochrome bc_1_ (complex III), and its exclusive use has driven resistance emergence in endemic regions (Barman et al., 2023). Rotenone acts on complex I of the host mitochondrial ETC—an entirely distinct node. This mechanistic divergence demonstrates that host-directed mitochondrial targeting can bypass parasite-intrinsic resistance mechanisms, a principle with broad implications for antiparasitic drug design (Yang et al., 2023). Drug combination studies extend this logic: strong synergy between Rotenone and BPQ (FIC = 0.2389 ± 0.12) and between Rotenone and atovaquone (FIC = 0.433 ± 0.084) demonstrates that co-targeting complexes I and III produces cooperative effects exceeding either agent alone. The activity of Piericidin A, a structurally distinct complex I inhibitor (Gutman et al., 1970), corroborates NADH dehydrogenase as a genuine pharmacological entry point, while the inactivity of 1-methyl-2-pentyl-4(1H)-quinolinone (Greco et al., 2025) confirms that complex I inhibition is not a uniform class effect and that structural specificity is required for productive target engagement. Taken together with the mitochondrial phenotypes observed, the Piericidin A results remain consistent with possible involvement of complex I, though further biochemical and genetic validation will be required to confirm target engagement and pathway-specific vulnerability in TA cells.

The cross-phylum activity of Rotenone constitutes an additional central finding. While prior studies have evaluated Rotenone and its derivatives against *Plasmodium falciparum* and *Leishmania panamensis* (Upegui et al., 2014), activity against *Leishmania donovani*, *Entamoeba histolytica*, and *Naegleria fowleri* has not previously been reported. Rotenone and its derivatives exert antiprotozoal effects through mitochondrial ROS induction and redox dysregulation rather than canonical complex I inhibition alone (Heinz et al., 2017), and the consistent activity observed here across representatives of Apicomplexa, Kinetoplastida, Amoebozoa, and Heterolobosea suggests that these evolutionarily distant organisms share conserved pathways linked to mitochondrial metabolism and oxidative stress regulation. The mechanistic basis becomes clear when each organism is considered in light of its mitochondrial biology. In apicomplexans, an intact ΔΨm is essential to sustain dihydroorotate dehydrogenase (DHODH) activity—the sole route for *de novo* pyrimidine biosynthesis and an established drug target (Triana et al., 2016). ΔΨm collapse therefore delivers a secondary metabolic lethal blow independent of direct complex I inhibition. In kinetoplastids such as *Leishmania*, with comparatively limited antioxidant defenses, the surge in mitochondrial superoxide is disproportionately damaging; Rotenone-induced ΔΨm disruption may additionally destabilize the kinetoplast, the highly organized mitochondrial DNA network essential to parasite survival. The activity against *N. fowleri* extends the reach of complex I inhibition to free-living pathogenic amoebae (Ženíšková et al., 2022), while the reduced sensitivity of *E. histolytica*—which lacks canonical mitochondria and harbors only non-respiring mitosomes (Mi-Ichi et al., 2015) —provides the critical mechanistic control, confirming that Rotenone’s antiprotozoal efficacy is intrinsically linked to functional mitochondria.The *L. donovani* assay was performed using the promastigote stage; therefore, these findings do not establish susceptibility of the clinically relevant intracellular amastigote. Future studies evaluating amastigote-stage activity will be required. For *N. fowleri*, data remain limited to a single concentration (20 μM), and concentration-response experiments will be necessary to determine an IC_50_.

This activity spectrum is further explained at the structural level by conservation of type II NADH dehydrogenase (NDH2) architecture. Protozoan parasites rely on NDH2 rather than mammalian-type complex I, and its absence in hosts has long positioned it as a parasite-selective target (Ke et al., 2019). Notably, however, the genetic dispensability of PfNDH2 during asexual blood-stage *Plasmodium falciparum* infection suggests that NDH2 alone does not account for Rotenone’s activity in apicomplexans, pointing instead to broader mitochondrial membrane potential disruption and downstream metabolic consequences as primary drivers of efficacy. Our comparative sequence, motif, and phylogenetic analyses nonetheless reveal a shared PTZ00318 superfamily domain architecture across *Plasmodium*, *Theileria*, *Babesia*, *Toxoplasma*, *Leishmania*, and *Naegleria*, while *Entamoeba* NDH2 diverges from this pattern—consistent with its loss of conventional mitochondrial machinery. This structural concordance aligns with the view that the NDH2 fold may represent an evolutionarily conserved scaffold (Gospodaryov, 2025) and positions the conserved PTZ00318 motifs as rational starting points for structure-guided inhibitor design. However, these observations are based solely on *in silico* analyses and do not establish direct Rotenone–NDH2 interaction or a functional role for NDH2 in susceptibility. Experimental validation involving genetic, biochemical, and structural approaches will be required to test this hypothesis.

## 4 Conclusion and future direction

The mechanistic framework established here opens several avenues for translating prototype activity into clinically relevant strategies. Structure-guided optimization of the Rotenone scaffold—building on analogs such as Rotenoisin A (Bak et al., 2021) —could yield inhibitors with improved selectivity and reduced systemic toxicity. Expanding structural diversity around this scaffold, informed by the transcriptomic and functional signatures defined here, could produce candidates suitable for *in vivo* evaluation. Systematic dissection of how inhibiting individual and combined ETC nodes affects parasite survival would define the hierarchy of bioenergetic dependencies and identify combination strategies with the lowest resistance risk. Finally, deeper characterization of parasite-specific mitochondrial interfaces—integrating metabolomic and proteomic datasets with the transcriptomic data generated here—would refine the model of host–parasite metabolic coupling, expose novel vulnerabilities beyond complex I, and provide the systems-level resolution needed to advance from mechanistic proof-of-concept to a tractable drug discovery programme.

## 5 Materials and methods

### 5.1 GHPB plate details

The Global Health Priority Box (GHPB) is a curated collection of 240 chemically diverse compounds organised into three sections, each containing 80 compounds targeting a specific global health priority area.

The MB2 (Drug-Resistant Malaria) section comprises 80 compounds selected by Medicines for Malaria Venture (MMV) and its partners, with confirmed activity against drug-resistant malaria parasites. The ZND (Zoonotic and Neglected Diseases) section contains 80 compounds derived from a Bristol-Myers Squibb compound library and selected for activity against neglected tropical diseases, zoonotic diseases, and pathogens at risk of developing drug resistance. The VEC (Vector Control) section includes 80 compounds chosen with input from experts at the Innovative Vector Control Consortium (IVCC) and evaluated for activity against various disease vectors. Together, these three sections provide a standardised resource for screening and identifying novel compounds with potential applications in antimalarial drug discovery, neglected disease research, and vector control strategies. The stock concentration in all the compounds provided is 10 mM.

### 5.2 *In vitro* drug sensitivity evaluation, compound screening, and cell culture

#### 5.2.1 Theileria annulata

*Theileria annulata–*infected bovine leukocyte cell line (TA cells) were maintained in RPMI‐1640 medium supplemented with 10% fetal bovine serum, 2 mM L‐glutamine, and 1% penicillin–streptomycin at 37 °C in a humidified 5% CO_2_ atmosphere. Buparvaquone (BPQ) was obtained from Sigma‐Aldrich (Cat# B4725). Peripheral blood mononuclear cells (PBMCs) from healthy cattle were used as controls for cytotoxicity assays (Barman et al., 2023). All experiments were performed in biological duplicates. The BPQ‐resistant *T. annulata* line (BPQ-R cells) was generated following (Singh et al., 2025).Briefly, parental cells sensitive to 200 ng/mL BPQ were exposed to gradually increasing concentrations, beginning at 10 ng/mL, until a population capable of sustained growth at ~10 µg/mL was established. The resistant phenotype remained stable even after drug withdrawal, with parasites continuing to proliferate in BPQ‐free medium for several weeks. This line was subsequently maintained under standard culture conditions and used for all resistance‐related assays.

#### 5.2.2 Leishmania donovani:

Promastigote-stage *Leishmania donovani* (Ag83 strain) parasites were maintained in Medium 199 supplemented with 10% heat-inactivated fetal bovine serum (FBS) under axenic conditions at 25 °C. Cultures were passaged to ensure late log-phase growth before the experimental setup (Devsani et al., 2023).

For drug susceptibility profiling, 1 × 10^5^ promastigotes per well were seeded into sterile 96-well flat-bottom plates containing two-fold serial dilutions of test compounds: Paromomycin (0.3–150 µg/mL), Amphotericin B (0.04–20 µM) and DMSO were used as positive controls. Plates were incubated for 48 hr at 25 °C under static conditions. Post-incubation, 50 µL of resazurin solution [0.0125% (w/v) in phosphate-buffered saline] was added to each well to assess metabolic activity. Plates were further incubated for 24 hr at 25 °C, and fluorescence intensity was measured using a microplate reader (excitation: 530–560 nm; emission: 590 nm) to determine cell viability and calculate IC_50_ values (Devsani et al., 2023).

#### 5.2.3 Entamoeba histolytica

Trophozoites of *Entamoeba histolytica* strain HM-1:IMSS were cultured axenically in BIS-33 medium supplemented with 15% (v/v) adult bovine serum (RM10913, HiMedia Labs, India), 2% (v/v) Diamond’s vitamin mix, 100 U/mL penicillin, 100 µg/mL streptomycin, and 0.25 µg/mL amphotericin B (Gibco, USA). Cultures were maintained at 35.5 °C under anaerobic conditions to support optimal trophozoite growth (Padavala and Verma, 2025)

For drug susceptibility assays, trophozoites were seeded at a density of 1.7 × 10^5^ cells/mL and exposed to the test compound for a duration exceeding 48 hr. DMSO-treated cells served as vehicle controls. On day 3 post-treatment, viable trophozoites were harvested and enumerated using a hemocytometer under phase-contrast microscopy to assess drug-induced effectivity.

#### 5.2.4 Naegleria fowleri

Trophozoites of *Naegleria fowleri* were cultured axenically in Nelson’s medium supplemented with 10% fetal bovine serum (FBS) at 37 °C under standard conditions. All experiments were conducted using logarithmic-phase trophozoites to ensure active proliferation and metabolic responsiveness (Güémez and García, 2021). For drug exposure studies, cells were seeded at a density of 1.9 × 10^5^ cells/mL and treated with Rotenone. DMSO (10 µL) was included as a vehicle control. At post-treatment, viable trophozoites were harvested and enumerated using a hemocytometer to quantify drug-induced effectivity. Representative images of treated cells were captured under bright-field microscopy to document morphological alterations.

### 5.3 Cell proliferation assay

To determine compound effects on cell proliferation, TA cells (1 × 10^6^ cells per well) were treated with the IC_50_ concentration of each drug and monitored over 48 hr. Cell viability and counts were assessed every 12 hr using the trypan blue exclusion method. Experiments were performed in triplicate, and mean values were plotted.

To provide an estimate of the selectivity of Rotenone toward the tested protozoan parasites, the selectivity index (SI) was calculated using the reported human-cell cytotoxicity value (LC_50_ = 378.8 ± 15.0 µM) obtained from the literature (Upegui et al., 2014), as we do not currently have access to the facilities required to determine Rotenone cytotoxicity in human cells. Although we evaluated Rotenone toxicity in healthy bovine PBMCs using a resazurin-based viability assay, no appreciable loss of viability or cell death was observed even at the highest concentration tested (100 µM), precluding reliable calculation of an LC_50_ and, consequently, an SI based on the bovine PBMC data. Therefore, the SI values presented here should be considered literature-based estimates intended to provide a comparative indication of the apparent parasite selectivity of Rotenone. The human-cell cytotoxicity value (378.8 ± 15.0 µM) is a literature-derived CC_50_/LC_50_ value and was used only to estimate the SI. For *N. fowleri*, an SI cannot be calculated because only a single concentration (20 µM) was tested and therefore an IC_50_ could not be determined (**Supplementary Figure 2B**).

### 5.4 Chemical similarity analysis

Chemical structures of MMV1828058, MMV692115, MMV002519, buparvaquone, and artesunate were obtained in SMILES format from PubChem and the MMV Pathogen Box database. Atom pair fingerprints (APfp) were generated using RDKit (v2023.03.1) in Python, and pairwise Tanimoto coefficients (Tc) were calculated to evaluate structural similarity, with values ranging from 0 (no similarity) to 1 (identical). The Tc matrix was subjected to hierarchical clustering analysis (HCA) using the (scipy.cluster.hierarchy) (Virtanen et al., 2020) module. A heatmap with dendrograms was generated using Seaborn to visualize structural diversity.

### 5.5 Western blotting

Western blot analysis was used to assess the temporal clearance of *Theileria* parasites post-treatment. Cell lysates dissolved in RIPA buffer were collected at 24 and 48 hr, and protein concentrations were measured. Proteins were separated by SDS-PAGE and transferred to membranes. Membranes were probed with primary antibodies, including rabbit anti-TaSP (1:1000), β-Actin (1:5000), Cleaved Caspase-3 (1:1000), Caspase-9 (1:2000), Caspase-8 (1:1000), Phospho-H2AX (Ser139) (1:1000), and HSP70 (1:1000), followed by appropriate HRP-conjugated secondary antibodies. Signals were detected using the Bio-Rad ChemiDoc™ imaging system and chemiluminescent HRP substrate.

### 5.6 Immunofluorescence analysis

Immunofluorescence assays were conducted to evaluate the subcellular localization and activation of H2AX (Ser139), p53, and cleaved Caspase-9. Treated cells were first washed twice with cold PBS to remove media and debris. Cells were then fixed with 4% paraformaldehyde (PFA) for 15 minutes at room temperature, followed by three washes with PBS. Fixed cells were permeabilized using 0.01% Triton X-100 in PBS for 15 minutes and subsequently blocked with either 5% goat serum or 3% BSA in PBS for 1 hr at room temperature to prevent nonspecific antibody binding.

After blocking, cells were incubated with the appropriate primary antibodies—Phospho-Histone H2AX (Ser139) (1:300), Phospho-p53 (Ser15) (1:500), and Cleaved Caspase-9 (1:800), overnight at 4 °C. Following incubation, cells were washed and treated with fluorophore-conjugated secondary antibodies (1:800) for 1 hr at room temperature in the dark. Nuclei were counterstained with DAPI. Imaging was performed using a Zeiss fluorescence microscope, and image processing was carried out using ZEN 3.3 (Blue Edition) software.

### 5.7 Apoptosis and cell death assays

Apoptotic and necrotic cell populations were assessed after 48-hr treatment with IC_50_ concentrations using Annexin V and propidium iodide (PI) staining. All assays were performed in duplicate to ensure reproducibility.

### 5.8 Cell cycle analysis

For analysis of cell cycle distribution, cells (1 × 10^4^/mL) were treated with VEC C04, ZND A06, and ZND D04 at their IC_50_ concentration and BPQ at 200ng/mL, fixed with ice-cold 70% ethanol, and stained with a PI-containing buffer (50 µg/mL PI, 0.1% Triton X-100, 200 µg/mL RNase) for 20 minutes at 37 °C. Samples were analysed using a BD flow cytometer, and data were processed using FlowJo X software.

### 5.9 TUNEL assay

DNA fragmentation was detected using the DeadEnd™ Fluorometric TUNEL System (Promega) according to the manufacturer’s protocol. Cells were treated with Rotenone for 24 hr, fixed with 4% paraformaldehyde, permeabilized with 0.1% Triton X-100, and incubated with TUNEL reaction mix for 1 hr at 37 °C. Nuclei were counterstained with DAPI, and fluorescence was observed using a Zeiss microscope. Quantification of TUNEL-positive cells was performed using ZEN 3.3 software.

### 5.10 Transcriptomic analysis (RNA-seq)

Total RNA was extracted using TRIzol (Invitrogen) and purified with the Qiagen RNeasy kit. RNA quality was verified using an Agilent Bioanalyzer. Libraries were constructed using the NEBNext Ultra II RNA Library Prep Kit and sequenced on an Illumina NovaSeq 6000 platform. Reads were aligned and quantified, and differential expression analysis was performed using DESeq2. Genes with an FDR< 0.05 and |log_2_ fold change| ≥ 1 were considered significant. Functional enrichment analysis was done using DAVID and GOseq, and protein–protein interaction networks were constructed using STRING v11.5.

### 5.11 Quantitative real‐time PCR

Total RNA was extracted from the respective cell samples using the Quick-RNA Microprep Kit (Zymo Research, R1051) according to the manufacturer’s instructions. One microgram of purified RNA was reverse‐transcribed into complementary DNA (cDNA) (Takara Bio, #6110A) using a first‐strand synthesis system optimised for full‐length transcripts. Quantitative real‐time PCR (RT‐qPCR) was performed with a SYBR Green–based detection chemistry, according to the supplier’s instructions, to measure relative transcript abundance. The host Actin gene served as the internal reference for normalisation of expression data. Primer sequences used in this study are listed in table (**Supplementary Figure 4**). Relative expression levels were calculated using the ΔΔCt method, and all reactions were performed in technical triplicates to ensure reproducibility.

### 5.12 JC-1 assay for mitochondrial membrane potential

Cells were incubated with 1 µM JC-1 dye (Thermo Fisher) at 37 °C for 20 minutes. After washing, red and green fluorescence intensities were measured by both fluorescence microscopy and flow cytometry (BD LSR Fortessa). Data were analyzed using FlowJo software to assess mitochondrial membrane polarization.

### 5.13 MitoTracker green assay for mitochondrial mass

Mitochondrial mass was assessed by staining cells with 100 nM MitoTracker Green FM (Invitrogen #M7514) for 30 minutes. After PBS washes, cells were imaged using a Zeiss fluorescence microscope. ZEN 3.3 software was used for quantification.

### 5.14 MitoSOX green assay for mitochondrial ROS

Mitochondrial superoxide levels were evaluated using 1 µM MitoSOX Green (Thermo Fisher #M36005) incubated with cells for 15 minutes at 37 °C. Cells were washed with PBS and immediately analyzed using flow cytometry and fluorescence imaging to quantify mitochondrial ROS.

### 5.15 DCFDA assay for intracellular ROS

To assess total intracellular ROS, cells were incubated with 20 µM DCFDA (Invitrogen) for 30 minutes at 37 °C. Fluorescence intensity was measured by flow cytometry, and ROS levels were expressed as fold-change relative to untreated controls.

### 5.16 EdU incorporation assay via click chemistry

Proliferation was analyzed using the Click-iT™ EdU Alexa Fluor™ 488 Imaging Kit (Thermo Fisher #C10337). Following treatment with IC_50_ concentrations for 48 hr, cells were pulsed with EdU for 3 hr, fixed, and permeabilized. The Click-iT reaction cocktail was added to detect incorporated EdU. DNA was stained with Hoechst, and images were acquired with a Zeiss fluorescence microscope at 63× magnification. Quantification was performed using ZEN 3.3 software.

### 5.17 Checkerboard drug combination assay

Stock solutions of Atovaquone (ATQ), Buparvaquone (BPQ), and Rotenone (ROT) were prepared in dimethyl sulfoxide (DMSO) and stored according to the manufacturers’ recommendations. Working solutions were freshly prepared by serial dilution in culture medium before each experiment. The highest concentrations used were 200 µg/mL (ATQ), 1000 ng/mL (BPQ), and 20 µM (ROT). Drug interactions were evaluated using a two-dimensional checkerboard assay in 96-well plates. Serial two-fold dilutions of each drug were prepared along orthogonal axes to generate a matrix of drug combinations, including ATQ + ROT, BPQ + ROT, and ATQ + BPQ. *Theileria*-infected cells were incubated with the drug combinations for 48 hr under standard culture conditions. Following incubation, cell viability was assessed using the resazurin reduction assay, and fluorescence was measured using a microplate reader at excitation/emission wavelengths of approximately 530–560 nm/580–590 nm. Percentage inhibition was calculated relative to untreated control cells.

Drug interactions were initially quantified using the fractional inhibitory concentration (FIC) index, calculated as:


$$FIC\;index=\frac{A}{MIC_{A}}+\frac{\text{B}}{MIC_{B}}$$

where *A* and *B* represent the concentrations of each drug in combination producing the inhibitory effect, and *MIC_A_*and *MIC_B_* represent the minimum inhibitory concentrations of each drug alone. Interactions were interpreted as:

• Synergistic: FIC< 0.5
• Additive/Indifferent: 0.5 ≤ FIC ≤ 4
• Antagonistic: FIC > 4

To further characterize drug interactions across the complete concentration matrices, the checkerboard datasets were independently analysed using the SynergyFinder platform. The drug-response matrices were analysed using four reference models: Highest Single Agent (HSA), Loewe additivity, Bliss independence, and Zero Interaction Potency (ZIP). Synergy scores were calculated across the concentration matrix, with positive scores indicating greater-than-expected combined activity and negative scores indicating less-than-expected activity according to the respective reference model. Statistical significance of the estimated synergy scores was assessed within the SynergyFinder analysis (Ianevski et al., 2017, 2022). Complete concentration–response matrices, model-specific synergy matrices, and corresponding heatmaps are provided in the **Supplementary Material**. Because different reference models are based on different assumptions regarding drug interactions, the results from the FIC analysis and SynergyFinder models were considered complementary measures of drug interaction rather than interchangeable estimates of synergy

Synergy scores were interpreted as:

• Positive values: synergistic interaction
• Negative values: antagonistic interaction
• Values near zero: additive effect

All experiments were performed in triplicate (n = 3), and data are presented as mean ± standard deviation (SD).

### 5.18 Dose–response and cytotoxicity assays for additional compounds

Piericidin A (PA) (#15379, Cayman Chemical) and 1-Methyl-2-pentyl-4(1H)-quinolinone (1MP4Q) (#TN1175, TargetMol) were obtained from commercial suppliers. Stock solutions of PA were prepared in 100% ethanol, while 1MP4Q was dissolved in DMSO. For dose–response analysis, both compounds were serially diluted starting from a maximum concentration of 100 µM. TA cells were treated with the compounds for 48hr, with ethanol- or DMSO-treated cells serving as vehicle controls. Cell viability was assessed using the resazurin assay, and IC_50_ values were determined from dose–response curves. To evaluate host cell toxicity, cytotoxicity assays were performed using peripheral blood mononuclear cells (PBMCs) isolated from healthy cattle. Cells were treated under similar conditions, and viability was measured using the resazurin assay to determine CC_50_ values. All experiments for these compounds were conducted in biological duplicates, and results are presented as mean ± SD.

### 5.19 Measurement of cellular ATP levels

Cellular ATP levels were measured using the ATP Colorimetric Assay Kit (E-BC-K157-S; Elabscience, USA) according to the manufacturer’s instructions. Briefly, cells were treated with Rotenone for 24 hr, after which 1 × 10^6^ cells from each experimental condition were collected. Cells were subjected to ATP extraction using 450 µL of ATP extraction solution. The extracts were processed according to the manufacturer’s protocol, and ATP levels were determined colorimetrically by measuring absorbance at 636 nm. ATP concentrations were calculated from a standard curve generated using the ATP standards provided with the kit. The cellular ATP content was expressed as nmol ATP per 10^6^ cells.

### 5.20 Statistical analysis

All experiments were conducted in biological duplicates unless stated otherwise. Statistical significance was assessed using unpaired two-tailed Student’s t-tests or one-way ANOVA, where appropriate. All data are presented as mean ± SD, with individual points representing biological replicates. Statistical significance was determined using the appropriate statistical test of unpaired t-test. ns, not significant; *p< 0.05; **p< 0.01; ***p< 0.001; ****p< 0.0001. For RNA-seq data, false discovery rate (FDR) was used to correct for multiple comparisons.

## Data Availability

The datasets presented in this study can be found in online repositories. The names of the repository/repositories and accession number(s) can be found below: https://www.ncbi.nlm.nih.gov/, PRJNA1475175.

## Supplementary material

Supplementary Figure 1 **(A–C)** Figures represents the Heatmaps generated with the help of Synergy Finder for all 3 drug combinations. **(D)** Figure represent the table with values generated for the checker board assay.

Supplementary Figure 2 **(A)** Bright field images of the Rotenone treated *N.floweri* parasites at different concentrations **(B)** Figure showing IC50 and SI values of Rotenone against different parasites. The human-cell cytotoxicity value (378.8 ± 15.0 µM) is a literature-derived CC_50_/LC_50_ value and was used only to estimate the SI. For *N. fowleri*, an SI cannot be calculated because only a single concentration (20 µM) was tested and therefore an IC_50_ could not be determined. ND: Not determined. NC: Not calculated.

Supplementary Figure 3 Sequence conservation, phylogenetic relationships, and motif architecture of NDH-2 homologs. **(A)** Multiple sequence alignment of representative NDH-2 protein homologs from *Theileria annulata* (TA05115), *Babesia bovis* (BBOV_I004980), *Plasmodium falciparum* (PF3D7_0915000), *Toxoplasma gondii* (TGME49_288830), *Naegleria fowleri* (NfTy_084590), *Leishmania donovani* (LdBPK_365620.1), and *Entamoeba histolytica* (EHI_139310). Conserved amino acid residues are highlighted in yellow, while highly conserved residues are indicated by colored shading. Alignment reveals strong conservation within the catalytic and cofactor-binding regions characteristic of alternative NADH dehydrogenases (NDH-2). **(B)** Maximum-likelihood phylogenetic tree constructed from full-length NDH-2 protein sequences. Branch lengths represent the number of substitutions per site, and bootstrap support values (%) are shown at major nodes. The analysis demonstrates the evolutionary relationships among apicomplexan and other eukaryotic NDH-2 homologs, with *Theileria parva* NDH-2 clustering most closely with the homolog from *T. annulata* (Dereeper et al., 2008) (Dereeper et al., 2008). **(C)** Conserved motif analysis of NDH-2 homologs performed using MEME. Colored boxes indicate the positions of conserved motifs within each protein sequence. Three highly conserved motifs were identified across most homologs, whereas variation in motif composition and distribution was observed in more divergent sequences. The corresponding motif consensus sequences are shown below the diagram. Statistical significance of motif enrichment is indicated by the associated E-values (Bailey et al., 2015). **(D)** Conserved domain superfamily details generated with help of CDD NCBI database.

Supplementary Figure 4 Table shows primers used in the study.
