# Supplementary material for: Complex I inhibition collapses host bioenergetics and activates p53‐dependent apoptosis in Theileria annulata infected cells
Source: Front Cell Infect Microbiol. 2026 Oct 19;16:1908136. doi: 10.3389/fcimb.2026.1908136 (PMC13724478; doi:10.3389/fcimb.2026.1908136)
Supplement: Supplementary file 1 [file SupplementaryFile1.pdf]

Supplementary Figure 1

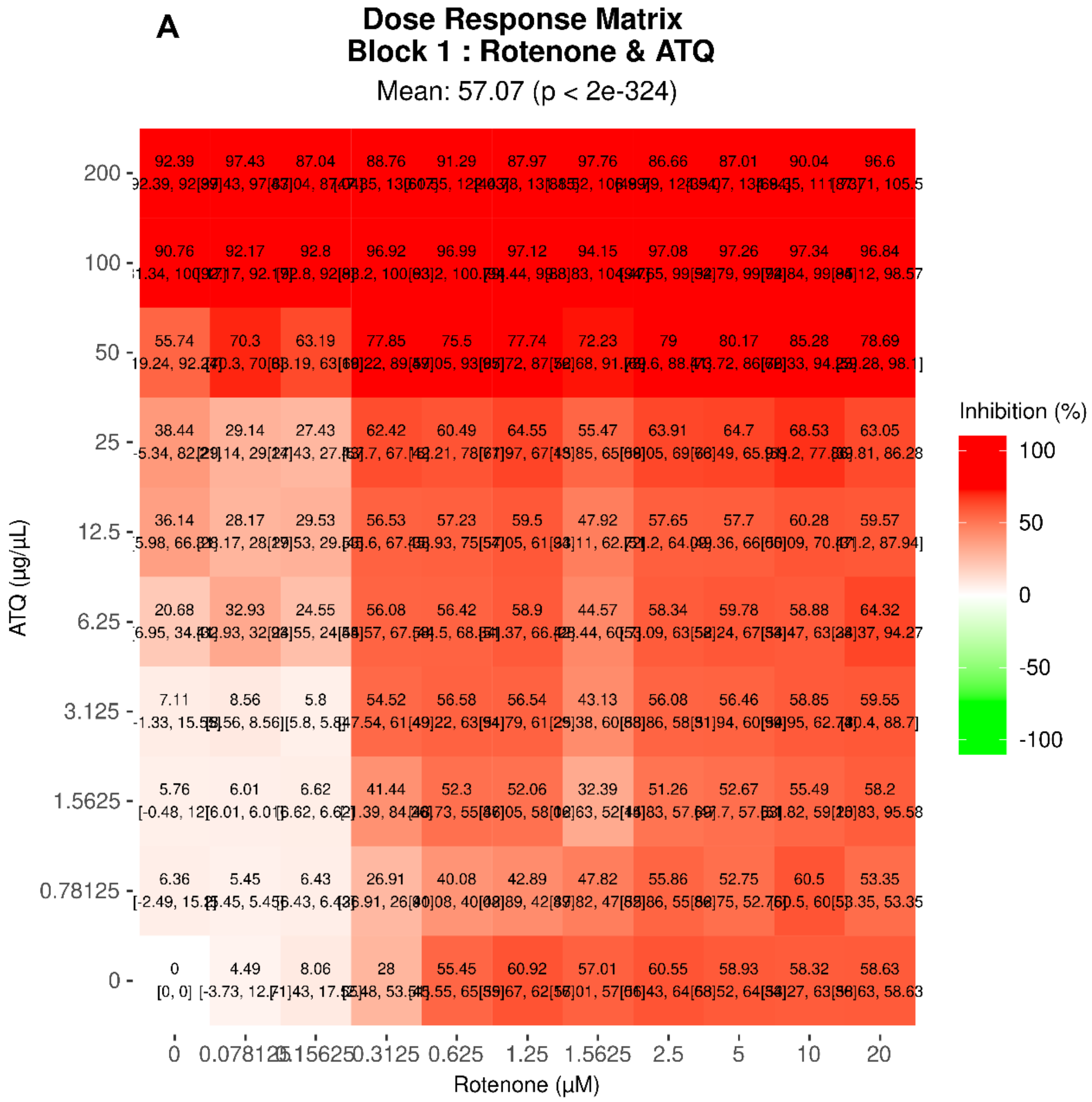

B

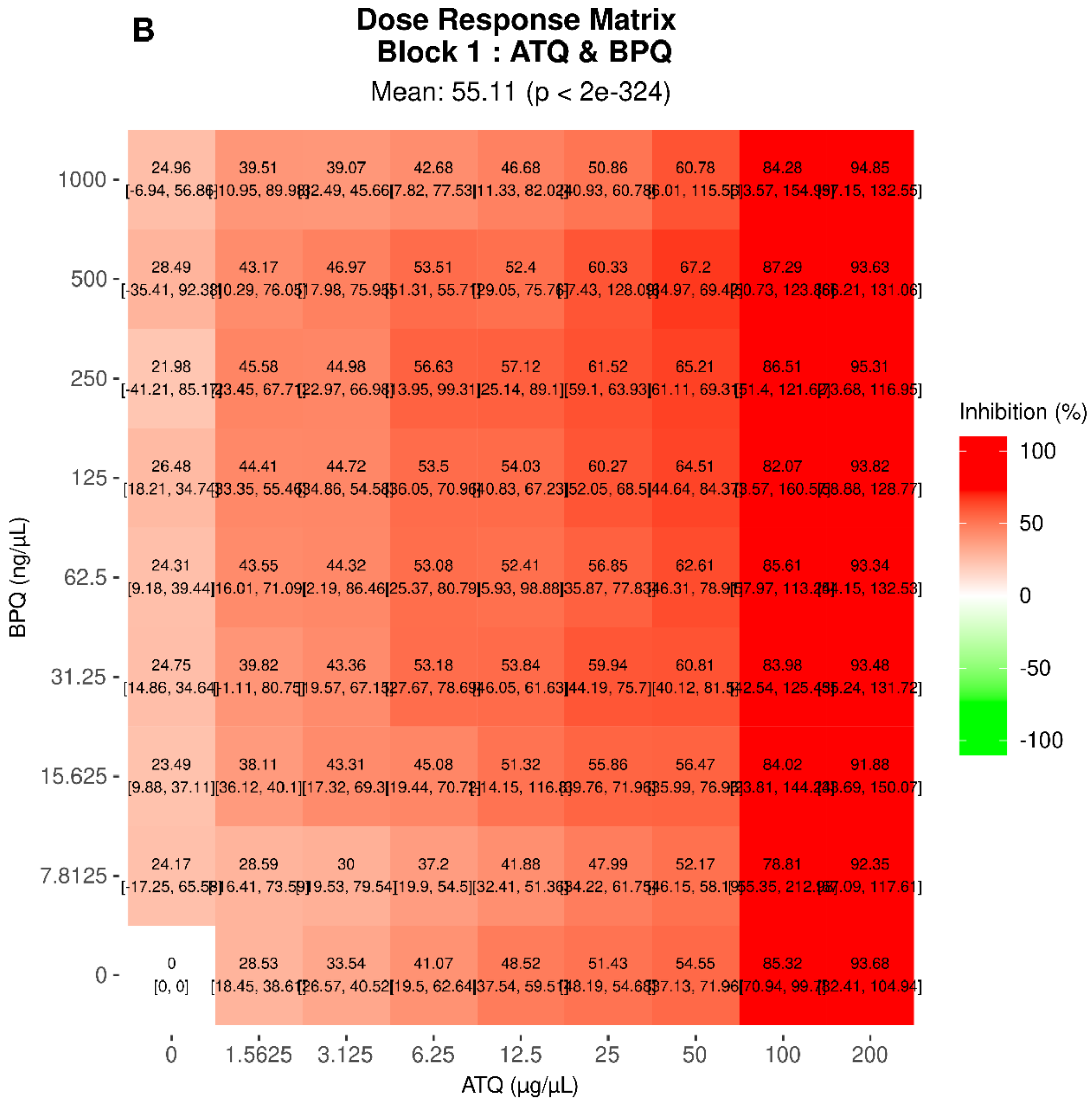

Dose Response Matrix
Block 1 : ATQ & BPQ
Mean: 55.11 (p < 2e-324)
Inhibition (%)
100
50
0
-50
-100
BPQ (ng/μL)
1000
500
250
125
62.5
31.25
15.625
7.8125
0
ATQ (μg/μL)
0
1.5625
3.125
6.25
12.5
25
50
100
200
24.96 39.51 39.07 42.68 46.68 50.86 60.78 84.28 94.85
28.49 43.17 46.97 53.51 52.4 60.33 67.2 87.29 93.63
21.98 45.58 44.98 56.63 57.12 61.52 65.21 86.51 95.31
26.48 44.41 44.72 53.5 54.03 60.27 64.51 82.07 93.82
24.31 43.55 44.32 53.08 52.41 56.85 62.61 85.61 93.34
24.75 39.82 43.36 53.18 53.84 59.94 60.81 83.98 93.48
23.49 38.11 43.31 45.08 51.32 55.86 56.47 84.02 91.88
24.17 28.59 30 37.2 41.88 47.99 52.17 78.81 92.35
0 28.53 33.54 41.07 48.52 51.43 54.55 85.32 93.68
[0, 0]

C

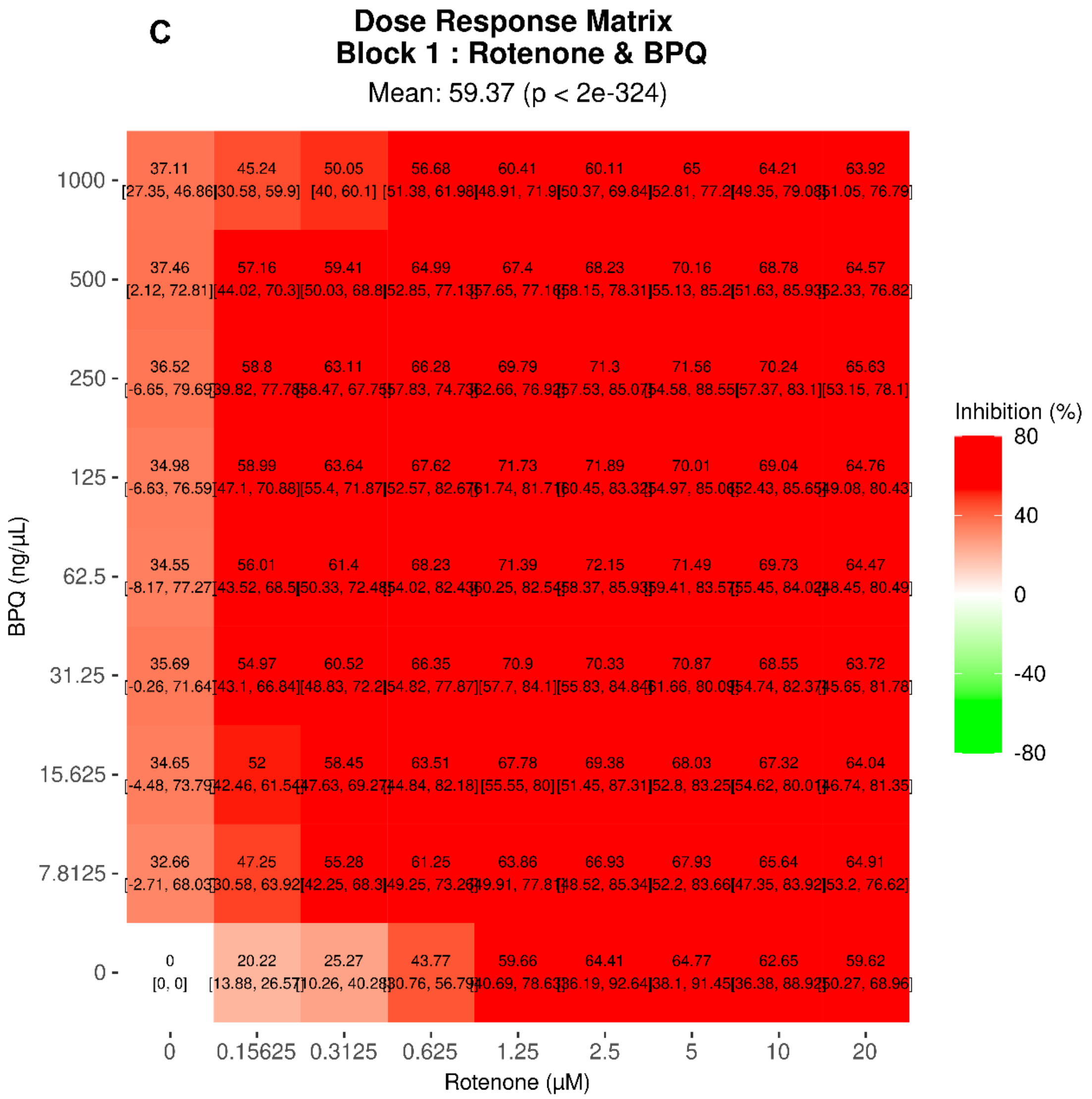

(D)

Drug combination	FIC index	HSA score	Loewe score	Bliss score	ZIP score
Rotenone + BPQ	0.239 ± 0.12	11.58	10.37	−2.68	−1.70
Rotenone + ATQ	0.433 ± 0.084	1.87	0.3	-5.52	-4.2
ATQ + BPQ	0.436 ± 0.020	5.48	5.51	−5.76	−5.44

(Note: FIC, fractional inhibitory concentration; HSA, Highest Single Agent; ATQ, atovaquone; BPQ, buparvaquone; ZIP, Zero Interaction Potency. FIC values were obtained from manually performed checkerboard assays. Synergy scores were calculated using SynergyFinder. Positive SynergyFinder scores indicate greater-than-expected combined activity, whereas negative

scores indicate less-than-expected combined activity according to the respective reference model.)

Supplementary figure 1 : **A, B and C Figures represents the Heatmaps generated with the help of Synergy Finder for all 3 drug combinations. (D) Figure represent the table with values generated for the checker board assay.**

Supplementary figure 2

(A)

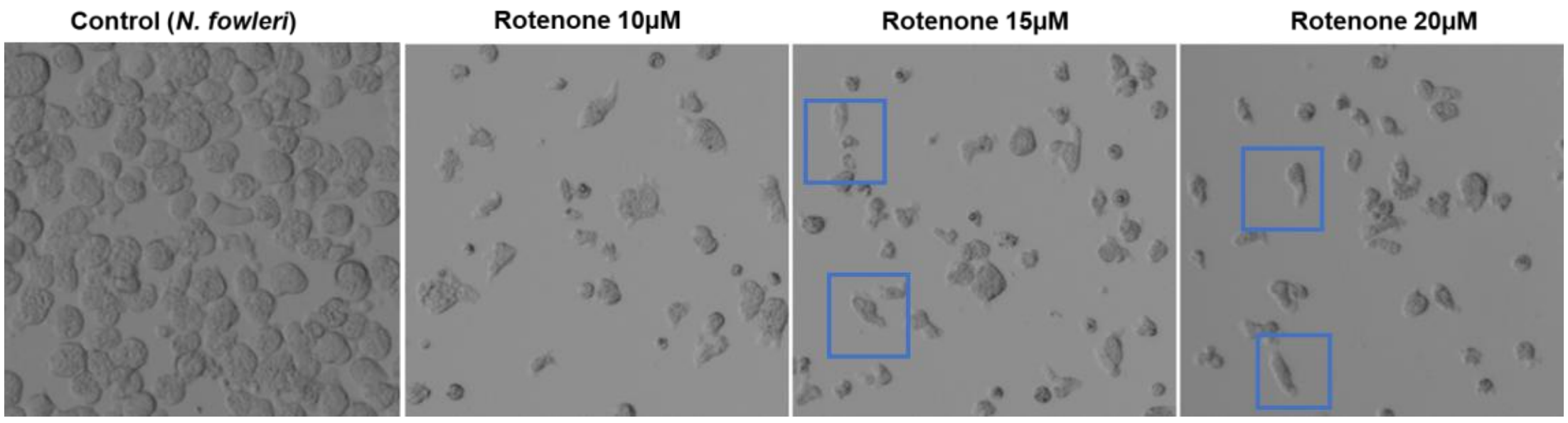

(B)

Parasite	Rotenone activity	IC_{50} / response (μM)	Reference human-cell CC_{50}/LC_{50} (μM)	SI (CC_{50}/IC_{50})
Theileria annulata	IC_{50}	**1.30 ± 0.27**	**378.8 ± 15.0**	**291.4**
Leishmania donovani	IC_{50}	**10.12 ± 0.98**	**378.8 ± 15.0**	**37.43**
Entamoeba histolytica	IC_{50}	**32.86 ± 0.32**	**378.8 ± 15.0**	**11.53**
Naegleria fowleri	Mortality at 20 μM	**44.27 ± 4.02% mortality**	**378.8 ± 15.0**	**NC**
**Parasite**	**Rotenone activity**	**IC_{50} / response (μM)**	**Bovine PBMCs from 100 μM**	**SI (CC_{50}/IC_{50})**
Theileria annulata	IC_{50}	**1.30 ± 0.27**	ND	**NC**

Supplementary figure 2:

(A): Bright field images of the rotenone treated *N.floweri* parasites at different concentrations **(B):** Figure showing IC50 and SI values of rotenone against different parasites. The human-cell cytotoxicity value (**378.8 ± 15.0 μM**) is a **literature-derived CC_{50}/LC_{50} value** and was used only to estimate the SI. For *N. fowleri*, an SI cannot be calculated because only a single concentration (20 μM) was tested and therefore an IC_{50} could not be determined. ND: Not determined. NC: Not calculated.

Supplementary Figure 3

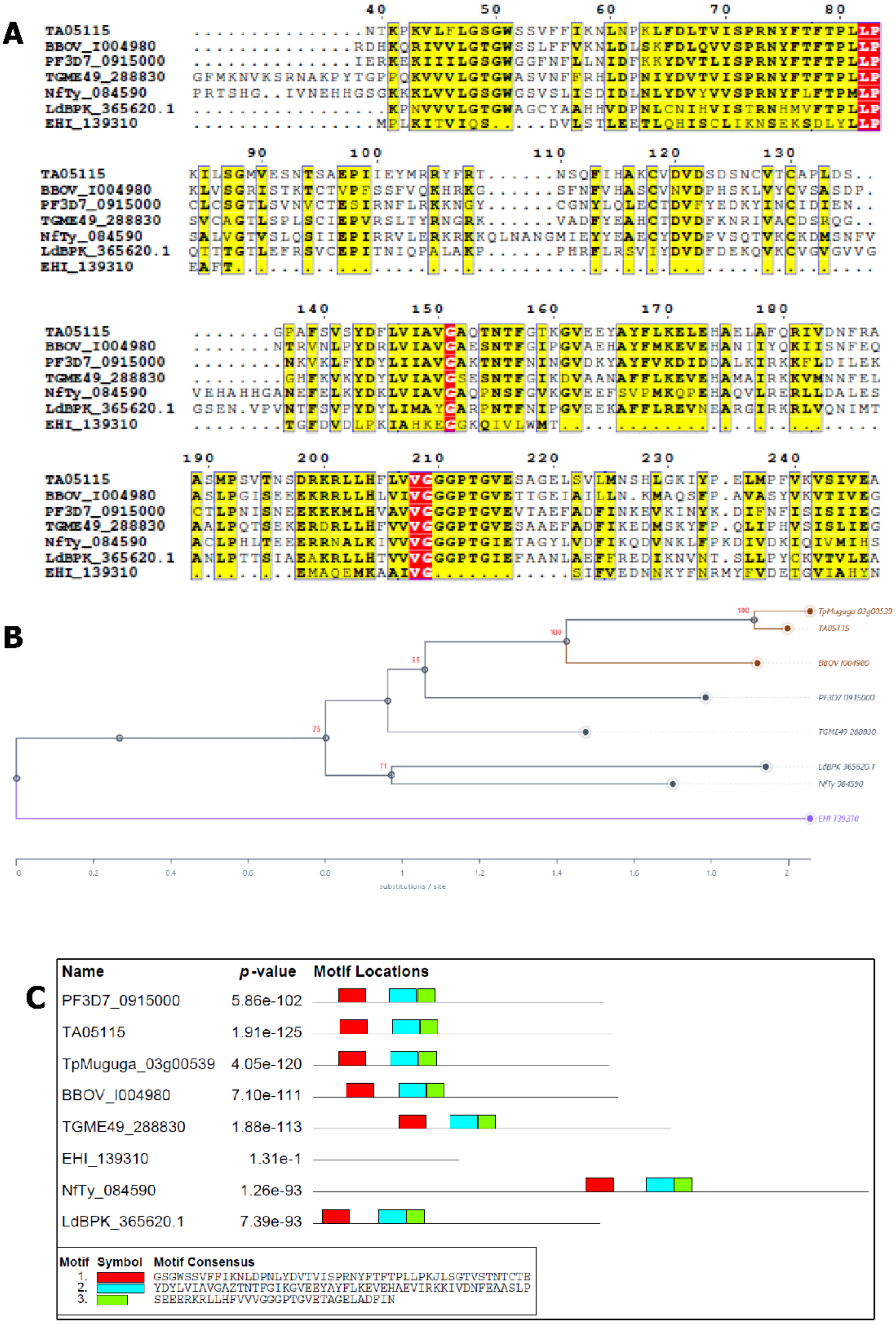

(D)

Protein Names	Organism	Conserved domain Superfamily
PF3D7_0915000	*Plasmodium falciparum*	PTZ00318
TA05115	*Theileria annulata*	PTZ00318
TpMuguga_03g00	*Theileria parva*	PTZ00318
BBOV_I004980	*Babesia bovis*	PTZ00318
TGME49_288830	*Toxoplasma gondii*	PTZ00318
LdBPK_365620.1	*Leishmania donovani*	PTZ00318
EHI_139310	*Entamoeba histolytica*	Nitrilase
NfTy_084590	*Naegleria fowleri*	FRQ1

Supplementary Figure 3. Sequence conservation, phylogenetic relationships, and motif architecture of NDH-2 homologs.

(A) Multiple sequence alignment of representative NDH-2 protein homologs from *Theileria annulata* (TA05115), *Babesia bovis* (BBOV_I004980), *Plasmodium falciparum* (PF3D7_0915000), *Toxoplasma gondii* (TGME49_288830), *Naegleria fowleri* (NfTy_084590), *Leishmania donovani* (LdBPK_365620.1), and *Entamoeba histolytica* (EHI_139310). Conserved amino acid residues are highlighted in yellow, while highly conserved residues are indicated by colored shading. Alignment reveals strong conservation within the catalytic and cofactor-binding regions characteristic of alternative NADH dehydrogenases (NDH-2).

(B) Maximum-likelihood phylogenetic tree constructed from full-length NDH-2 protein sequences. Branch lengths represent the number of substitutions per site, and bootstrap support values (%) are shown at major nodes. The analysis demonstrates the evolutionary relationships among apicomplexan and other eukaryotic NDH-2 homologs, with *Theileria parva* NDH-2 clustering most closely with the homolog from *T. annulata*. (Dereeper et al., 2008)(Dereeper et al., 2008)

(C) Conserved motif analysis of NDH-2 homologs performed using MEME. Colored boxes indicate the positions of conserved motifs within each protein sequence. Three highly conserved motifs were identified across most homologs, whereas variation in motif composition and distribution was observed in more divergent sequences. The corresponding motif consensus sequences are shown below the diagram. Statistical significance of motif enrichment is indicated by the associated E-values. (Bailey et al., 2015).

(D) Conserved domain superfamily details generated with help of CDD NCBI database.

Supplementary Figure 4. Table shows primers used in the study

Name	Sequence
qBT-Actin - For	GGCATCCTGACCCTCAAGTA
qBT-Actin - Rev	CACACGGAGCTCGTTGTAGA
HSPA6_For	CGAGCCCGCTTTGAAGAACT
HSPA6_Rev	GCTCCCTGCCATCGAAGAAG
G2E3 For	AACTCTGTGCAACCCTATGG
G2E3 Rev	CCAATGCAGGTAGACGTAAGAG
GPNMB For	GTGCATCTCCTGACCTGTATG
GPNMB Rev	TTGTCACTGAACACTGTCCC
NDUFA4L2 For	GCAGATCAAGAGACATCCCG
NDUFA4L2 Rev	GGAACTTGTATTGGTCATTGGG
DNAJB1 For	GCCGAAATCCCTTTGACAAC
DNAJB1 Rev	CTCTTCAAGTGAGACCCTAAGG
DHTKD1 For	CGAGCCCTTCTTCACCAACC
DHTKD1 Rev	CCTGGGTTGTGAGTCCGTTG
ERBB3 For	CGGGAAGGCACCCTTTCTTC
ERBB3 Rev	GGGAGGCACTGAGGTCTGAT
GARS1 For	CCCTGCCGTAGTAGCTCCAT
GARS1 Rev	CCTGGTCAAGGCCTCCGATA
RB1 For	ACAAGCAAAGGACCGAGAAG
RB1 Rev	GTCTGGAAGGCTGAGGTTG
EIF1 For	TCAACAGAGAAACGGCAGG
EIF1 Rev	GTCCAATCTCTACCAGGAACTG
EIF5 For	TTAATCGGCCTCCAACGTATC
EIF5 Rev	GGATTCTCACACTCAGGACAG
EEF1E1 For	AACAATGGTCCGAGTCTAACG
EEF1E1 Rev	GTCATCTTTACTGGAGTGTCCG
